# Design of an Interactive Mind Calligraphy System by Affective Computing and Visualization Techniques for Real-Time Reflections of the Writer’s Emotions

**DOI:** 10.3390/s20205741

**Published:** 2020-10-09

**Authors:** Chao-Ming Wang, Yu-Chen Chen

**Affiliations:** Department of Digital Media Design, National Yunlin University of Science and Technology, Douliu 64002, Taiwan; k.1209@hotmail.com

**Keywords:** affective computing, brain wave, calligraphy, emotion, human–computer interaction, mental state, visualization

## Abstract

A novel interactive system for calligraphy called mind calligraphy that reflects the writer’s emotions in real time by affective computing and visualization techniques is proposed. Differently from traditional calligraphy, which emphasizes artistic expression, the system is designed to visualize the writer’s mental-state changes during writing using audio-visual tools. The writer’s mental state is measured with a brain wave machine to yield attention and meditation signals, which are classified next into the four types of emotion, namely, focusing, relaxation, calmness, and anxiety. These emotion types then are represented both by animations and color palettes for by-standing observers to appreciate. Based on conclusions drawn from data collected from on-site observations, surveys via Likert-scale questionnaires, and semi-structured interviews, the proposed system was improved gradually. The participating writers’ cognitive, emotional, and behavioral engagements in the system were recorded and analyzed to obtain the following findings: (1) the interactions with the system raise the writer’s interest in calligraphy; (2) the proposed system reveals the writer’s emotions during the writing process in real time via animations of mixtures of fish swimming and sounds of raindrops, insects, and thunder; (3) the dynamic visualization of the writer’s emotion through animations and color-palette displays makes the writer understand better the connection of calligraphy and personal emotions; (4) the real-time audio-visual feedback increases the writer’s willingness to continue in calligraphy; and (5) the engagement of the writer in the system with interactions of diversified forms provides the writer with a new experience of calligraphy.

## 1. Introduction

**Figure 1:**
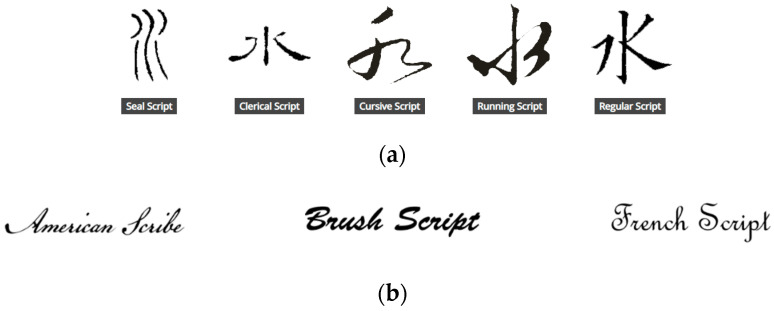
Calligraphy styles and script typefaces. (**a**) Five basic styles in oriental calligraphy (from left to right): seal script, clerical script, cursive script, running script, and regular script [2]. (**b**) Some script typefaces of western languages [5].

Calligraphy is a thousands-of-years-old art, which helps express the human’s perception of the world through aesthetic writing of strokes [1,2,3]. It is traditionally presented on a piece of paper with a soft hair brush or a pen in certain basic styles [4], and may be regarded as the originator of a great many of fonts or typefaces used daily in the digital era. Some calligraphy styles used in oriental languages are the seal script, clerical script, cursive script, running script, and regular script [2], as shown in Figure 1a; and some script typefaces used in western languages [5] are shown in Figure 1b.

With the advances of the human–computer interaction technology, the integration of digital art with calligraphy provides a new form of presentation and interaction between people via digital devices [3]. For instance, a haptic interface device using a tablet with a pen can be used to obtain a calligraphy experience different from that obtained via the conventional way conducted by a calligrapher or by a learner of calligraphy [6]. However, the calligraphic works using the haptic interface device might seem to be lacking human emotions. Poria et al. [7] claimed that human emotions can be recognized, felt, inferred, and interpreted by intelligent systems using the affective computing technology which is regarded as part of the recent development of human–computer interaction technologies. Additionally, several studies of utilizing human beings’ continuous brain or physiological signals to detect personal emotions have been proposed recently and used for many applications successfully [8,9,10,11,12].

In view of these advancements, it can be said that new technologies have brought calligraphy into a new era in two aspects. One is to use a touch panel on a desk top as a good helper to overcome the difficulty of motion expressions via the use of hard-pens and keyboards, and the other is to use a brain wave machine to measure the emotional information. In addition to these two aspects, the technology of visualization, which is any technique for creating images, diagrams, or animations to communicate a message, can be employed to visualize the writer’s artistic expression and inner affection in the process of calligraphy [4]. Therefore, it is desirable and significant to design an affective computing-based interactive system for calligraphy which satisfies the requirements of the above three aspects of system design.

In this study, a new human–machine interfacing system, called mind calligraphy, for the above-mentioned purpose of calligraphy, which can intelligently reflect the emotional state of the participant in the writing process by affective computing and visualization techniques, is proposed. Specifically, while a participating writer is using the proposed system to perform calligraphy, his/her brain waves are measured continuously to reflect his/her emotions in real time, which are classified into four types, namely, focusing, relaxation, calmness, and anxiety. Each of the emotion types is then transformed, by visualization techniques, into various forms of multimedia, including images, sounds, and animations. Finally, the visualization results are displayed or played using a set of specially-designed interactive devices for inspection by the writer himself/herself, the system designer, and by-standing observers.

In addition to developing the hardware architecture of the proposed system, various kinds of evaluation of the system’s performance and the participating writers’ experiences in using the system via public exhibitions, questionnaire surveys, interviews, and audio-visual recordings were also conducted to prove the usefulness of the proposed system. Two main research issues are addressed in these multi-dimensional evaluations of the proposed “mind calligraphy” system. (1) What is the writer’s affection in the calligraphy revealed by the proposed system? (2) What is the writer’s interactive behavior in using the proposed system? These issues are answered in this study via the aforementioned systematic evaluations of the system and are described in detail subsequently.

In the remainder of this paper, a review of the related studies, including a literature review of existing methods and an introduction to the techniques involved in the proposed system, is given in Section 2. Then, the methodology for designing the proposed system based on affective computing is presented in Section 3. The details of the processes in constructing the proposed system are presented in Section 4. The adopted methods for evaluating the system performance from various viewpoints are described in Section 5, followed by the presentation of the experiments conducted in this study and the evaluations of the system’s performances in Section 6. Some concluding remarks and discussions are found in Section 6. Some more details of the system’s construction and performance evaluations are presented in the appendices.

## 2. Related Studies

In this section, a survey of related works is given at first, followed by a review of the techniques involved in the proposed system, including those for affection expressions and brain wave measurement for affective computing in calligraphy.

### 2.1. A Survey of Related Works

**Figure 2:**
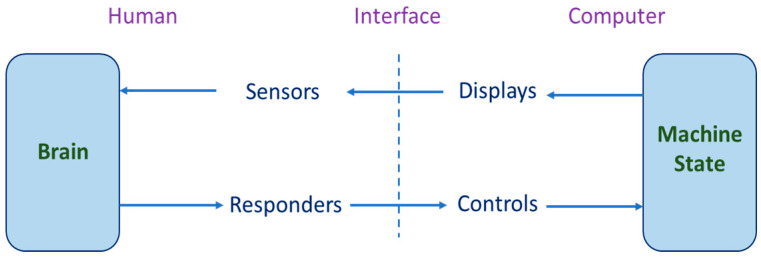
The human-factor view of a human operator in a human-computer interfacing working environment [13].

With the progress of the computer and digital processing technology, the study of interaction between the human being and the computer via human–computer interfacing devices is getting more and more versatile and diversified. Kantowitz and Sorkin [13] proposed a concept of human–computer interfacing from the viewpoint of human factor, which says that a human being has sensors and responders while the computer consists of displays and controls; when the human being senses a display on the computer screen, he/she will give responses through the brain and then control the computer, as shown in Figure 2.

The study of affection is a complicated domain involving a wide range of research fields, and studies from different fields giving various definitions for the term “affection.” Schachter [14] believes that affection is a label used to connect an individual with a certain physical state based on a general hypothesis, and that the affection describing an individual refers to cognitive factors and physiological arousals.

Apart from the definition, the classification of affection is also a focus of related academic studies. Ping [15] classified the expressions of affection into four categories, namely, happiness, anger, fear, and sorrow, and Ekman [16] proposed six types of human affection according to the human facial expressions, namely, joy, distress, anger, fear, surprise, and disgust.

In addition, reflecting the rapid technological developments that resolve a lot of problems in people’s daily lives, Picard [17] proposed the term “affecting computing” in 1995, expecting to bring forth affection to the computer. Generally speaking, a system designed for use in the study of affecting computing will include various sensors to obtain the expressive or physiological signals caused by human affection, analyze the signals and related data, identify the human affection, and finally yield proper responses.

### 2.2. Ways for Affection Expressions in Calligraphy

Kao [18] asserted that writing is an expressive behavior of a writer and is directly affected by his/her emotions. Similarly, calligraphy, which is based on meaningful aesthetic stroke creation, not only can be conducted to express a calligrapher’s or a calligraphy beginner’s writing style but also can allow him/her to express his/her emotion. In other words, besides being useful for expressing character meanings or for generating artistic decorations, calligraphy tends to reveal a deep and subconscious function created by the writer’s heart. It shows the writer’s personal characteristics and reflects his/her mental state.

Therefore, in addition to the exploration of the style and modeling of calligraphy, the affective information left in the calligraphic work is also inseparable and worth a deep investigation. One can learn the emotion of a calligrapher via not only the written characters, but also the handwriting style. Sassoon [19] thought that the way of writing indicates the mental status of a person at that moment, and is the visible trace left by handwriting and the external outcome left by the body and mind. A deep analysis of the calligraphic work “Draft of a Requiem to My Nephew” by Chen-Ching Yan, a famous calligrapher in ancient China, revealed the fact that Yan suffered from the utmost pain of the loss of a beloved relative [20]. By observing Yan’s calligraphic work, it can be found that he wrote the characters swiftly and corrected them repeatedly, using the clear edges and corners of the characters and the light and heavy strengths of them to reveal the ups and downs of his emotions, respectively. It all means that calligraphy unfolds a very strong interrelationship between the written strokes and the affection at the time in the writing process.

### 2.3. Brain Wave Measurement in Affective Computing

Affective computing covers many subjects for investigations, such as cognition science, medicine, machine learning, signal processing, computer vision, sensor design, user-oriented interfacing, and so on. It aims to make a computer respond to its user appropriately and to handle the relation between the user’s emotion and the computer suitably [21,22]. In this aspect, Picard and Klein [23] divided the studies in the affective computing field into four major types, namely, recognizing emotions, expressing emotions, having emotions, and conveying emotional intelligence; and Yang [24] suggested the adoption of the brain wave from which physiological signals can be extracted to represent various types of emotion. About the measurement of the brain wave, Berger [25] first recorded the electrical activities of the human brain and used them the term electroencephalogram (EEG), from which the brain wave can be obtained. Aftanas et al. [26] classified the brain wave into four categories, namely, delta (0–4 Hz), theta (4–8Hz), alpha (8–13 Hz), and beta (13–30 Hz).

Along with the advances of brain science and technology, the brain wave has been utilized as a type of interface signal to control machines in the past two decades, just like the function of a keyboard for a computer, resulting in a hot research topic called brain–computer interfacing (BCI). It was mentioned by scholars participating in the First Brain Computer Interface Conference [27] that the study of BCI provides a person with an alternative channel to communicate with the outside with no need to rely on the human’s peripheral nerves and muscles as the media.

Considering the EEG being useful for direct reflections of humans’ emotional states with relatively low costs and simplicity, Kim et al. [28] reviewed the computational methods that have been developed to deduct EEG indices of emotion, to extract emotion-related features, or to classify EEG signals into one of many emotional states; mentioned that EEG-based emotional state estimation requires well designed computational methods to extract information from complex and noisy multichannel EEG data; and proposed the use of sequential Bayesian inference to estimate the continuous emotional state in real time. Furthermore, Rieiro et al. [29] conducted an in-depth comparison of the EEG recording qualities of a medical-grade device, the SOMNOwatch + EEG-6, and a consumer-grade device, the NeuroSky MindWave Mobile [30]; and revealed the fact that though the MindWave Mobile is noise-limited, it provides stable recordings even through long time periods with its data being of adequate quality compared to that of conventional wet electrode EEG devices, except for a potential calibration error and spectral differences at low frequencies.

In this study, the brain wave device NeuroSky MindWave Mobile was adopted to detect a person’s brain waves. The brain waves were then processed via some algorithms of the so-called eSense Meters built into the device to yield two mental states, namely, attention and meditation, of the writer. The two kinds of mental state were taken as pattern features in this study to classify the calligrapher’s emotions into the four types, namely, focusing, relaxation, calmness, and anxiety, as mentioned previously.

## 3. The Methodologies for Designing the Proposed System Based on Affective Computing

The methodologies taken for designing the proposed system are described in detail in this section, including the planning of the research process for developing the proposed system, the collections of ideas for designing the system from the literature review, and the interviews with three experts in related fields.

### 3.1. The Research Process for System Development

The research process adopted in this study for designing and developing the proposed system included the following major steps:

(1) collecting preliminary design ideas before the system was designed—both from the literature review and from a series of interviews with invited experts of affective computing;
(2) designing a prototype system—based on the collected preliminary design ideas;
(3) testing the prototype system in a public exhibition—to collect opinions from the system designer and the participating writers for improving the prototype system;
(4) improving the prototype system into a formal system—according to the opinions collected from the last step;
(5) testing the formal system in a second exhibition—to collect more opinions from people like those in Step (3); and
(6) evaluating the system performances and the participating writers’ experiences—according to the opinions collected in the last step.

The aforementioned activities conducted in the adopted research process can be seen to provide a systematic way of designing, exhibiting, and improving the development of the proposed affective computing system for calligraphy whose usages and performances are different from those of the traditional calligraphy approach.

### 3.2. Ideas for System Design Based on the Literature Review

Based on the literature review conducted in this study, as described previously, the following set of ideas is proposed for designing the proposed system:

(1) recording the calligraphy process performed by the writer using a writing brush;
(2) measuring the mental states of the writer with a brain wave machine;
(3) utilizing digital tools as the means for human–machine interfacing to offer a new experience of calligraphy to the participating writer; and
(4) converting the measured mental states of the writer into emotions which are then expressed by animations and sounds.

### 3.3. Ideas for System Design Based on Interviews with Experts

The interview method is a qualitative approach adopted by many research domains in which the dialogue between an interviewer and an interviewee is utilized to find the ideas, motives, and attitudes of the two sides [31]. In this study, interviews with three experts, one being a calligrapher and the other two specialists in the human–computer interaction field, have been conducted to collect ideas for designing the proposed system. The questions asked in the interviews included three aspects: (1) the significance of calligraphy; (2) the way to apply interaction techniques to calligraphy; and (3) the relation between calligraphy and human emotions. The three experts’ comments were collected and summarized in the following:

(1) the system to be developed should combine the modern technology and life elements;
(2) a brain wave instrument should be used to collect the brain signals of the writer;
(3) the brain signals should be converted into types of emotion; and
(4) the system should be unique and can indeed present the emotions of the writer interactively by visualization techniques.

These comments were taken as another set of ideas followed in designing the system process and in refining the content of the questionnaire for this study which is described in the next section.

## 4. Construction of the Proposed System

In this section, after describing the process followed in this study for constructing the proposed system, the architecture, interaction devices, and interaction mechanism of the system are presented, followed by a description of a method proposed in this study for classification of the writer’s emotions using the mental-state features provided by the brain wave machine mentioned previously. Two applications of the classification results, namely, color palette construction and animation generation, are also finally proposed.

### 4.1. Development Process and Design Concepts of the Proposed System

To construct a calligraphy system with human–machine interaction functions for this research, as pointed out by Eliason [32], the system development process should include the following four steps:

(1) proposing the interactive demands and items for the design of the interaction devices in the system;
(2) constructing the system to include an emotion recognition module, an interactive feedback mechanism, and a hardware device;
(3) integrating and testing the software and hardware for the interaction mechanism; and
(4) exhibiting the constructed system and evaluating its performance.

The above major steps of interactive system development were followed in the design of the proposed system, as elaborated in the following.

It is difficult for a person other than the calligrapher to understand the emotions which are expressed by the writer during the writing process and hidden in the writing result. We attempted in this study to solve this issue by designing a calligraphy system using affective computing techniques to visualize the writer’s emotions in real time. Calligraphy with a function of emotional visualization is termed “mind calligraphy” in this study, as mentioned before. Moreover, the interaction process performed by the proposed system is designed to include the following three parts: (1) allowing the writer to experience calligraphy; (2) visualizing the changes of the writer’s emotions; and (3) displaying the calligraphic works created by the writer. More about those parts will be described in detail subsequently.

### 4.2. The Architecture and Interactive Devices of the Proposed System

**Figure 3:**
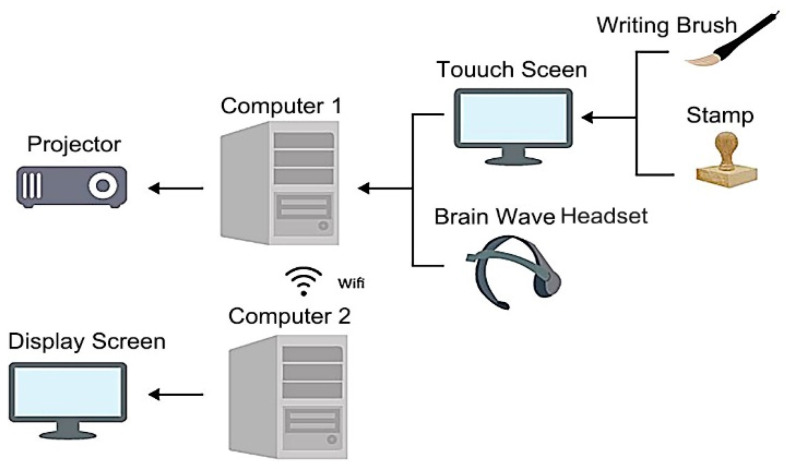
The architecture of the proposed system.

**Figure 4:**
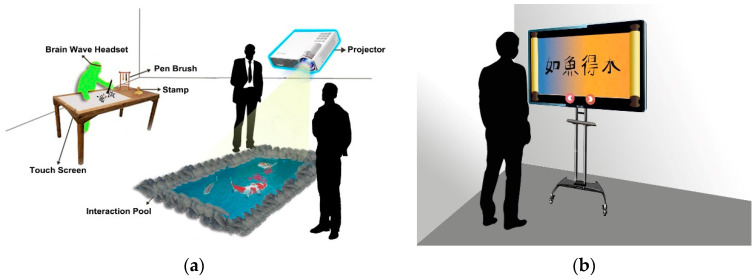
An illustration of the interaction environment and the devices of the proposed system. (**a**) The touch screen at the server site and the interaction pool at the client side. (**b**) The display screen at the remote site, on which the calligraphic work with a color-palette background is shown.

**Figure 5:**
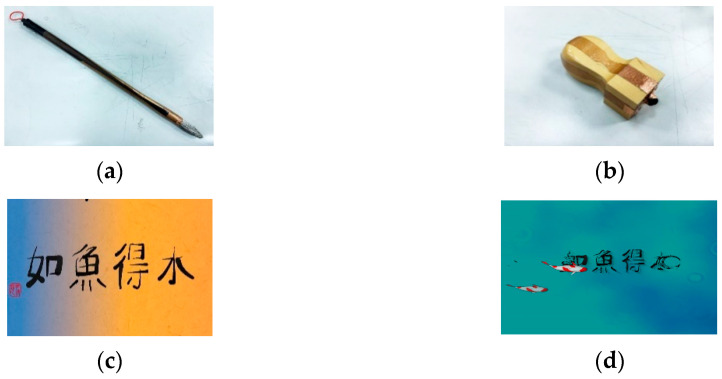
Tools used by the proposed system and calligraphic works with color-palette and animation backgrounds created in the interaction process. (**a**) The pen brush. (**b**) The stamp. (**c**) A calligraphic work with a color-palette background shown on the touch screen (with the four characters meaning “like a duck to water”). (**d**) A calligraphic work with an animation background shown on the panel of the interaction pool.

The architecture of the proposed “mind calligraphy” system is shown in Figure 3; the interaction environment using the system is illustrated in Figure 4; and two tools used by the system and two typical completed calligraphic works with “emotion-corresponding” backgrounds generated in the interaction process are shown in Figure 5.

As shown in Figure 3, the input devices of the system include a capacitive touch screen affixed on a table and a brain wave machine put on the writer’s head. The machine is a brain wave headset named MindWave Mobile manufactured by the NeuroSky, Inc. mentioned previously. The touch screen is used as the writing interface, allowing the writer to use a pen brush to conduct calligraphy and to perform user interfacing operations. The pen brush was specially-designed in this study and is shown in Figure 5a. A stamp, also designed in this study and shown in Figure 5b, is used to create a red seal on a completed calligraphic work by clicking on a pre-defined area on the screen. It is noted, by the way, that stamping a red seal on a painting or a calligraphic work is a common practice of oriental artists originating from ancient times.

The brain wave machine is used to measure the writer’s mental state continuously, based on which corresponding visual and audio feedback, including “emotion-corresponding animations” and “images of completed calligraphic works with emotion-reflecting backgrounds,” are generated by the proposed system, as will be explained in detail later in this section. Additionally, a projector hung on the ceiling is used to project the animations and the images of completed works onto the panel of an interaction pool at a client side for a bystander to observe.

When the writing is finished, one computer used as the server of the proposed system at the server site will transmit the writer’s calligraphic work in real time via a WiFi-based network to another computer of the system at a remote site, which then shows the work on a display screen for other bystanders to observe. Furthermore, an “advance icon” on the display screen can be touched to browse the displayed works.

Regarding the interaction environment using the proposed system as illustrated in Figure 4, more details of the man–machine interaction activities occurring during the calligraphy process are described in the following from the respective views of the interaction devices.

(1) The touch screen at the server site

On the touch screen on the working table shown in Figure 4a, there exist three buttons of the functions “select”; “send”; and “remove.” The writer may touch the button “select” to choose a set of characters from a copybook to practice calligraphy, as mentioned previously. Through a touch on the button of “send,” the calligraphic work can be projected onto the panel of the interaction pool at the client side for the by-standers there to observe. As for the third button of “remove,” it is used to eliminate a completed calligraphic work on the screen in order to start another writing session. Additionally, a color palette consisting of strips with colors reflecting the writer’s emotions at the time is created every 20 s; the details for this will be described subsequently.

When a calligraphic work is completed, the writer will use the stamp to click on the touch screen to create a red seal on the left edge of the work, as mentioned before, and the system will send the result to the server of the system. Furthermore, the system will merge the color palette as a background with the stamped calligraphic work to create a “formal” work (as shown in Figure 5c), which is sent finally to the display screen at the remote site to show to the by-standing observers, as illustrated by Figure 4b.

(2) The interaction pool at the client side of the system

The brain wave signals of the writer measured with the brain wave machine are classified into four types of emotion, as mentioned previously, based on which different animations (including audio and visual components of colored water, animated fishes, raindrops, and thunder to be described subsequently) are generated every five seconds by multimedia techniques as the feedback and are projected onto the interaction pool to show, with the associated sounds being played by a built-in speaker inside the projector.

Additionally, when the “send” button on the touch screen is pushed, the current calligraphic work is sent to the interaction pool to show in the animation being played continuously there.

(3) The display screen at the remote side

As mentioned previously, each “formal” calligraphic work consisting of a completed calligraphic work, a red seal, and a color-palette background sent from the server site is shown on the display screen at the remote site for by-standers there to inspect, as shown in Figure 4b. Such formal works are kept in the system server in sequence, and can be selected by an observer to appreciate by touching the “advance icon” on the display screen, as mentioned previously.

### 4.3. Emotion Classification and Applications to Color Palette and Animation Generations

Here, a scheme proposed in this study for emotion classification is described first. Then, two applications of the scheme, namely, color palette generation and animation creation, are presented with their corresponding algorithms described in detail.

#### 4.3.1. Classification of Emotions

The instrument used for brain wave measurement in the proposed system, as mentioned previously, is the MindWave Mobile headset developed by the NeuroSky, Inc., which provides 512 brain wave samples every second in the form of two mental-state signals, namely, attention and meditation. Therefore, each brain wave sample may be regarded as a two-feature vector. The proposed system was designed to collect such sample vectors every 20 s from a participant conducting calligraphy using the proposed system, and classify the sample vectors into the aforementioned four types of emotion, namely, focusing, relaxation, calmness, and anxiety. The criteria proposed in this study for this emotion classification process are described by a set of decision rules described by Equations (1) and shown in Table 1 with the additional specifications of colors for the emotion types, where the two variables *a* and *m* represent the features of attention and meditation, respectively.

(1) $$\left\{ \begin{array}{lll} a\geq 70\;\mathrm{and}\;a\geq m & \Rightarrow & \mathrm{focusing};\\ m\geq 70\;\mathrm{and}\;m>a & \Rightarrow & \mathrm{relaxation};\\ 30\leq a<70\;\mathrm{and}\;30\leq m<70 & \Rightarrow & \mathrm{calmness};\\ a<30\;\mathrm{or}\;m<30 & \Rightarrow & \mathrm{anxiety}. \end{array}\right.$$

The rules described above were derived from a decision-rule learning process conducted in this study as described in the following.

**Figure 6:**
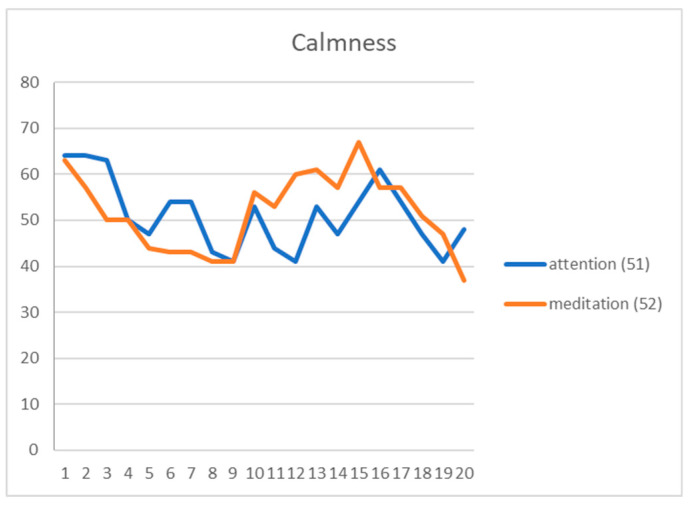
The sample sequence of the two-feature sample sequences measured by the brain wave device of the emotion type of calmness with average feature values of attention *a* = 51 and meditation *m* = 52 (note: the average feature values *a* and *m* satisfy the decision rule for calmness, namely, 30 ≤ *a* < 70 and 30 ≤ *m* < 70).

**Figure 7:**
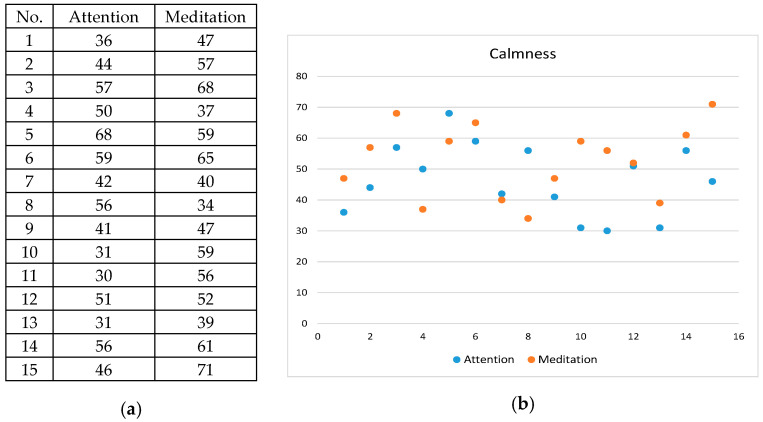
The data and the distribution graph of the average values of the 15 measured two-feature sample sequences of the emotion type of calmness from which the corresponding decision rule 30 ≤ *a* < 70 and 30 ≤ *m* < 70 for emotion classification is inferred, where *a* and *m* represent the attention and meditation signals, respectively. (**a**) The average data. (**b**) The graph.

(1) Four guests were invited to participate the learning process by wearing the brain wave device NeuroSky MindWave Mobile to generate brain wave signals while using the proposed mind calligraphy system.
(2) Four types of activity were designed for the participant to perform to arouse respectively the four types of emotion of focusing, relaxation, calmness, and anxiety as listed in the following:
  (a) reciting a well-known poem in one’s mind—to arouse the emotion of focusing;
  (b) listening to a light musical melody while vision focusing on a fixed spot—to arouse the emotion of relaxation;
  (c) watching a video clip playing quiescent scenes—to arouse the emotion of calmness; and
  (d) watching a video clip of horror movies—to arouse the emotion of anxiety.
(3) The guests were asked to conduct, for each type of emotion, the corresponding activity listed above to generate at least three 20-s sample sequences of the two-feature signals of attention and meditation, giving us a total of 15 sample sequences for the four types of emotion, with each sequence including 20 signals (i.e., taking one sample every second).
(4) At the end of the process of measuring each participant’s sample sequences for each emotion type, the participant was asked to take a rest of at least 15 min before the second measurement was started, for the purpose of preventing the participant from getting tired of the measurement and yielding imprecise signal data.
(5) An example of sample sequences so measured is shown in Figure 6 in which the averages (51 and 52) of the sample sequence values of the two features are also shown at the right.
(6) Data measurements of each emotion conducted on the four participants were carried out under the same conditions; i.e., the participants were all asked to perform the same activity (e.g., to listen to an identical light musical melody for arousing the emotion of relaxation).
(7) Furthermore, after each emotion data measurement was completed, the involved participant was interviewed to see whether he/she was in a stable or right mood or not during the measurement process; if not, the measured data were discarded because the measurement task is part of the learning process of inferring the ranges for emotion-type classification and erroneous data would cause incorrect inferred ranges.
(8) As an example, the distribution graph of the 15 average values of the two-feature sample sequence data of attention and meditation of the emotion type of calmness is shown in Figure 7. This graphs and those of the other three emotion types are shown in Appendix A.

#### 4.3.2. Generation of Color Palettes

The above scheme of emotion classification has been applied in this study to two tasks: color palette generation and animation creation, which have been mentioned previously. For the first application, at first four colors, namely, blue, green, orange, and aubergine, are used to represent the four types of emotion, namely, focusing, relaxation, calmness, and anxiety, respectively, as shown in the third column in Table 1. Additionally, within every 20 s, the colors of the sequence of classified emotion types are collected, summed up, and transformed into a color palette according to the following algorithm (described by pseudo-code). Each color palette is of a rectangular shape consisting of four-color strips corresponding to the four types of emotion, respectively, with the width of each strip being proportional to the ratio of the number of the corresponding colors with respect to the total number of sample colors collected in the 20-s period. We recall, by the way, that the color palette is used as the background of a completed calligraphic work and sent to the remote site to show on the display screen for by-standers to observe, as shown in Figure 4b.

**Algorithm 1** Generation of a color palette**Input:** a sequence of *n* brain wave samples *s*_1_, *s*_2_, …, *s_n_* in a duration of 20 s with each *s_i_* consisting of the two features of attention and meditation represented by variables *a_i_* and *m_i_*, respectively, i.e., *s_i_* = (*a_i_*, *m_i_*).**Output:** a color palette *H* consisting of four strips of colors of blue, green, orange, and aubergine from left to right representing the emotion types of focusing, relaxation, calmness, and anxiety, respectively.
**Steps.**

**Step 1:**

*//Initialization*
 1.1 denote the four emotion types of focusing, relaxation, calmness, and anxiety by *F_i_, R_i_, C_i_,* and *A_i_*, respectively; 1.2 set up four counters *F*_total_, *T*_total,_
*C*_total_, and *A*_total_ for *F_i_, R_i_, C_i_,* and *A_i_*, respectively.
**Step 2:**

*//Classifying the brain waves*
 **for**
*i* = 1 **to**
*n*
**do**  (a) classify *s_i_* = (*a_i_*, *m*_i_) as one of *F_i_, R_i_, C_i_,* and *A_i_* according to rules specified by Equation (1), and denote the result by *X_i_*;  (b) increment the counters according to *X_i_* in the following way:
*//Computing sums*
   (i) **if**
*X_i_* = *F_i_*, **then** set *F*_total_ = *F*_total_ + 1;    **end if;**
*//F_total_ = # blue samples*
   (ii) **if**
*X_i_* = *R_i_*, **then** set *R*_total_ = *R*_total_ + 1;    **end if;**
*//R_total_ = # green samples*
   (iii) **if**
*X_i_* = *C_i_*, **then** set *C*_total_ = *C*_total_ + 1;    **end if;**
*//C_total_ = # orange samples*
   (iv) **if**
*X_i_* = *A_i_*, **then** set *A_t_*_otal_ = *A*_total_ + 1;    **end if;**
*//A_total_ = # aubergine samples*
 **end for.**
**Step 3:**

*//Computing the color ratios and transforming them into 0~3*
 compute the following integers as the color weights:  *f* = ⎣(*F*_total_/*n*) × 4⎦, *r* = ⎣(*R*_total_/*n*) × 4⎦; *c* = ⎣(*C*_total_/*n*) × 4⎦; and *a* = ⎣(*A*_total_/*n*) × 4⎦.
**Step 4:**

*//Creating the color palette*
 4.1 create a rectangular shape *H* of size 4*w* × *h* pixels;
*//The unit width w and unit height h are pre-determined*
 4.2 fill *H* with blue-, green-, orange-, and aubergine-colored strips of the widths of *f*×*w*, *r*×*w*, *c*×*w*, and *a*×*w* pixels, respectively; 4.3 smear the boundary between every two different colors in *H* to create a color gradient effect within a distance of (1/3)×*w* from the boundary.
*//Making the created color palette look more natural*

**Step 5:**

*//Ending*
 exit with *H* as the desired color palette.

The operation “smearing the boundary” mentioned in Step 4.3 of Algorithm 1 aims at enhancing the visual effect of the entire output of the emotion type. Specifically, we add a color gradient stripe segment to the boundary of every two neighboring colors in the output. For four types of emotion, there are 35 different combinations according to Equation (2):

(2) $$C_{4}^{7}=\frac{7!}{3!4!}=35$$

Accordingly, 35 distinct gradient segments have been prepared in advance and kept in a database for use by table look-up operations when needed during the boundary-smearing process.

Some examples of the color palette created by Algorithm 1 are shown in Table 2. For example, the first color palette appearing in the second row of the table is composed of three-color strips with color weights 0, 2, 1, and 1 for the emotions of focusing, relaxation, calmness, and anxiety, respectively, where 0 means that the corresponding emotion does not appear in the 20-s duration. Each color palette so generated will, if sent out as part of a “formal” calligraphic work (as described in Section 4.1), be shown on the display screen as the background of a completed calligraphic work at the remote site. An observer there could see from the color palette the mixture of the writer’s emotions in the last 20 s before the calligraphic work was completed.

#### 4.3.3. Generation of Animations

About the animations mentioned above, Table 3 shows a list of the scenarios of the animations designed in this study to represent respectively the four types of emotion: focusing, relaxation, calmness, and anxiety. In addition, all the writer’s emotions in the duration of every five seconds are collected, and the one which appears for the most of the time is selected as the representative emotion in the five-second period and a corresponding animation is then projected on the panel of the interaction pool for a by-stander to observe. The scenario of each animation consists of a mixture of four types of animation effect as described in the following:

(1) the color shown on the pool background—including one of the four colors of blue, green, orange, and aubergine, which represent the aforementioned representative emotions;
(2) the number of raindrops—including one of three categories of “a small number,” “a large number,” and “a huge number”;
(3) the direction and speed of fish movement—including the four categories of “in the same direction with a medium speed,” “up and down with a slow speed,” “in different directions with a medium speed,” and “in chaotic directions with a fast speed”; and
(4) the sound types of played audio—including the four types of “raindrop,” “insect,” “river,” and “thunder.”

For example, for the emotion of focusing, the corresponding scenario is described in the second column of the last row in the table, which includes the following three parts of audio-visual presentations representing three meanings about the emotion:

(1) “fishes swim in the same direction with a medium speed”—meaning that the writer is focusing his/her mind on writing;
(2) “a small number of raindrops fall”—meaning that the writer is paying a certain degree of attention to writing; and
(3) “the raindrops sound clear”—meaning that the writer seems to have a very strong intention and his/her mind is concentrated.

In addition, colors are used to represent the writer’s emotions and are shown as the pool background. For example, the blue color is adopted in this study to represent the emotion of focusing. The descriptions of the scenarios corresponding to all of the four types of emotion are listed in the last row of Table 3 with the corresponding animation effects described in rows 2 through 5 in the table. It is noted again that the type of animation is determined by that of the emotion of the writer, which in turn is the result of classification of the mental-state features provided by the brain wave measurement machine.

The above discussions about the generations of emotion-representing animations may be described in detail as an algorithm in the following.

**Algorithm 2** Generation of an emotion-representing animation**Input:** a sequence of *n* brain wave samples *s*_1_, *s*_2_, …, *s_n_* sent to the server in a duration of 5 s with each sample *s_i_* consisting of the two features of attention and meditation represented by the variables *a_i_* and *m_i_*, respectively, i.e., *s_i_* = (*a_i_*, *m_i_*).
*//The input is the same as that of Algorithm 1 except that the time duration is 5 s instead of 20 s*
**Output:** an animation with its scenario being composed by multimedia techniques according to Table 3, representing one of the emotion types of focusing, relaxation, calmness, and anxiety, which appears most frequently during the period of 5 s.
**Steps.**

**Step 1:**

*//Initialization*
 1.1 denote the four emotion types of focusing, relaxation, calmness, and anxiety by *F_i_, R_i_, C_i_,* and *A_i_*, respectively; 1.2 set up four counters *F*_total_, *T*_total,_
*C*_total_, and *A*_total_ for *F_i_, R_i_, C_i_,* and *A_i_*, respectively.
**Step 2:**

*//Classifying the brain waves*
 **for**
*i* = 1 **to**
*n*
**do**  (a) classify *s_i_* = (*a_i_*, *m*_i_) as one of *F_i_, R_i_, C_i_,* and *A_i_* according to rules specified by Equation (1), and denote the result by *X_i_*;  (b) increment the counters according to *X_i_* in the following way:
*//Computing sums*
   (i) **if**
*X_i_* = *F_i_*, **then** set *F*_total_ = *F*_total_ + 1;    **end if;**
*//F_total_ = # blue samples*
   (ii) **if**
*X_i_* = *R_i_*, **then** set *R*_total_ = *R*_total_ + 1;    **end if;**
*//R_total_ = # green samples*
   (iii) **if**
*X_i_* = *C_i_*, **then** set *C*_total_ = *C*_total_ + 1;    **end if;**
*//C_total_ = # orange samples*
   (iv) **if**
*X_i_* = *A_i_*, **then** set *A_t_*_otal_ = *A*_total_ + 1;    **end if;**
*//A_total_ = # aubergine samples*
 **end for.**
**Step 3:**

*//Finding the emotion appearing most frequently*
 3.1 find the maximum of *F*_total_, *R*_total_, *C*_total_, and *A_t_*_otal_ and denote it by *M*_total_; 3.2 let the emotion type corresponding *M*_total_ be denoted as *T*_total_.
**Step 4:**

*//Creating the desired animation*
 create an animation *A* of the emotion type *T*_total_ according to the scenario listed in Table 3 by multimedia techniques.
**Step 5:**

*//Ending*
 exit with animation *A* as the desired output.

About the operation “create an animation by multimedia techniques” mentioned in step 4 of Algorithm 2 above, like what we did to smear color boundaries, we prepared in advance four distinct animations according to the scenario descriptions listed in Table 3, and used them via table look-up operations when needed. The Autodesk Maya software package was used to create the animations in advance instead of rendering them online. Therefore, the animations could be played in real-time.

### 4.4. An Algorithm of the Interaction Mechanism of the Proposed System

The process of the interaction mechanism performed by the proposed system described in the previous two sections, Section 4.2 and Section 4.3, may be presented more clearly as an algorithm in terms of pseudo-code in the following, where it is assumed that the brain wave headset is already put on the calligrapher’s head. Additionally, it is noted that when the aforementioned three buttons on the touch screen are pushed, the corresponding signals labeled “select,” “send,” and “remove,” respectively, will be issued by the system. Furthermore, as the stamp is used to click on the pre-set area on the touch screen to generate a red seal on the completed calligraphic work, a signal labeled as “seal” will be issued by the system. These signal labels will be used in the following algorithm, which is presented from the system’s operational point of view.

**Algorithm 3** The interaction mechanism of the proposed system
**Input:**
 (1) a blank space on the touch screen with three buttons of the functions of “select,” “send,” and “remove”; (2) a sequence of 512 *brain wave samples* every second in the forms of *attention and meditation signals* provided by the brain wave machine; (3) a digital copybook of calligraphy with a sequence of sets of characters.
**Output:**
 (1) a series of animations which are played on the panel of the interaction pool to reflect the emotions of the writer every 5 s; (2) the same as (1) above but additionally with the calligraphic work being superimposed on the animation in a floating manner as shown in Figure 5d when the calligraphic work is sent out by pushing the button of ‘send’; (3) a completely calligraphic work with a red seal and a color-palette background (created dynamically every 20 s) as shown in Figure 5c which is sent to show on the display screen as shown in Figure 4b when a writing session is ended by pushing the stamp on the touch screen to create the red seal; (4) the same as that of (3) above but sent instead to the server of the proposed system.
**Steps.**

**Step 1:**

*//Starting a new writing session*
 1.1 if the signal of “select” is detected, **then**   show the next set of characters in the digital copybook of calligraphy
*//Allowing the writer to choose a desired set of characters*
  **end if;** 1.2 show the result of character-set selection on the touch screen.
**Step 2:**

*//Looping while calligraphy is in progress*
 **while** the signal of “seal” is not detected **do**:  2.1 allow the writer to use the pen brush to conduct calligraphy and show the calligraphic work on the touch screen;  2.2 **for** every 20 s **do:**   (a) **for** every 5 s **do:**
*//Generating and plays animations*
    (i) perform Algorithm 2 with the brain wave samples as the input to generate an animation *A*    (ii) play *A* by projecting it onto the interaction pool to show the visual content and using the built-in speaker to play the associated sound;    **end for;**   (b) **if** the signal of ‘send’ is detected **then**
*//Showing the written work on the interaction pool*
    (i) superimpose the current calligraphic work *W*_0_ in a floating manner in the currently-played animation *A* to create a new animation *B*;    (ii) play *B* by projecting it onto the interaction pool to show the visual content and using the built-in speaker to play the associated sound;    **end if;**   (c) perform Algorithm 1 with the brain wave samples as the input to create a color palette *P*;   (d) **if** the ‘seal’ signal is detected **then**
*//Showing the work on the display screen at the remote site*
    (i) superimpose a red seal on the pre-set area on the left side of the current calligraphic work *W*_0_ to create a new work *W*_1_;    (ii) send *W*_1_ to the cloud server of the system to keep *W*_1_ there;    (iii)superimpose *W*_1_ on the color palette *P* to create a second new work *W*_2_;    (iv) send *W*_2_ to the display screen at the remote site to show;   **end if;**  **end for;** **end while.**
**Step 3:**

*//Ending or starting another writing session*
 **if** the signal of ‘remove’ is detected **then**   clear the completed calligraphic work on the touch screen and go to Step 1;
*//Starting another writing session*
  **else**   **if** the signal of ‘turn off system’ is not detected **then**    go to Step 2;
*//Keeping the writing session*
  **else** exit;
*//Ending*
 **end if;**
**end if.**


To illustrate further the interaction mechanism of the proposed system described by Algorithm 3, an example of illustrating the results of performing the algorithm is shown in Table 4, in which the intermediate results, including the animations and the calligraphic works, yielded by the major steps in the algorithm are shown, making the details of the algorithm easier to understand.

## 5. Introduction to Adopted Methods for Evaluations of the Proposed System

In this study, three approaches were adopted for evaluating the goodness of the proposed calligraphy system from the viewpoints of system usability and writer’s behavior, experience, or affection. The first approach was to use the observational method [33], the second to conduct questionnaire surveys, and the third to carry out interviews. The approaches are introduced in this section and the results of their uses for evaluations of the collected data in these approaches are described in the next section.

### 5.1. The Observational Method for Evaluating System Usability and the Writer’s Behavior

The observational method introduced in Lidwell et al. [34] was adopted in this study to conduct observations of the two indicators of system usability and writer’s behavior. The observations were conducted while the writer was interacting with the system to perform calligraphy. A qualitative scheme of the method was used to collect the data from the observations.

Specifically, at first the research purpose and the system operation procedure were introduced to the writer. Then, the observation of the writer’s performance in the calligraphy process was conducted by audio-visual recording, note taking, image acquisition, etc. The detailed items of the two indicators proposed in this study for such observations are listed in Table 5.

### 5.2. Questionnaire Design for Evaluating System Usability and Writers’ Experiences

Questionnaires can be used to collect desirable data effectively in various surveys [35]. A questionnaire must include simple and concise descriptions of the items to be evaluated, in order to avoid guiding the participating writer overly so that the data in the questionnaire can be effective. The items listed in the questionnaire used in this study were designed for the two indicators of system usability and writer’s experience about his/her interactions with the system. In doing this, the following considerations have been addressed.

(1) Consideration of the indicator of system usability

Nielsen [36] have proposed the following five indicators for evaluating system usability: (1) ease to learn; (2) efficiency; (3) ease to memorize; (4) error rate; and (5) satisfaction to analyze the usability. Norman et al. [37] assumed that a system with good usability should cover the following six items: (1) visibility; (2) feedback; (3) limitation; (4) correspondence; (5) consistency; and (6) prediction. Yeh [38] pointed out three indicators to evaluate the usability of an interactive design: (1) effectiveness; (2) ease; and (3) enjoyability. These studies can be seen to have similar concepts, based on which 12 questions were designed in this study—as shown in the upper part of the third column of Table 6—for use in the questionnaire for evaluating the usability of the proposed system.

(2) Consideration of the indicator of writer’s experience

When the proposed system is used by a writer, the major man–machine interaction includes the calligraphy action which may cause the writer’s real-time emotional changes and the audio-visual feedback of the proposed system, which is in various multimedia forms (animations, images, sound, etc.). The ideas involved in the strategic experience modules proposed by Schmitt [39], which cover five types of experience about the writer’s affections, namely, sensory experience, affective experience, creative cognitive experience, social-identity experience, and behavior and lifestyle, were found in this study to be pertinent to the exploration of the writers’ feelings in this interaction process and their opinions on the system usage. Based on these types of experience, ten questions were designed for use in the questionnaire for evaluating the writer’s experience, as listed in the lower part of the third column of Table 6.

In addition, the Likert 5-point scale [40] was adopted for use in the questionnaire to give the scores of 5 to 1 to each question in the questionnaire, which correspond to the opinions of “strongly agree” to “strongly disagree,” respectively.

Finally, after the questionnaire data from all the writers were collected completely, the software packages of SPSS and AMOS were adopted to find groups of related questions listed in Table 6, with each group having a common property called question dimension (or scale). The latent question dimensions so found of the two indicators, system usability and writer’s experience, are specified in Table 6 as well. The details of this process of finding such latent dimensions of the questions are described in Appendix B.2 in Appendix B.

### 5.3. Interview with the Writers

According to Kuan et al. [41], in the procedure of a face-to-face interview with a respondent, a researcher asks questions and the interviewee answers the questions to tell about their feelings and thoughts. More specifically, the researcher prepares an outline of the interview focusing on a topic, lists the main questions, and then uses a one-on-one style to carry out the interview; our theme focused on understanding the interviewee’s feelings and thoughts. The design of the questions is based on an outline; and depending on the interview situation, adjustments of different open-ended questions are conducted. At last, the respondent is guided to make an in-depth description on the topic.

In this study, a semi-structural interview was adopted according to the above-mentioned main points of the interview procedure. In short, the interview outline designed for uses in the study included the following indicators: (1) operation of the man–machine interfacing mechanism; (2) opinion on the calligraphy procedure; and (3) opinion on the affective computing process. The detailed items based on the above three indicators, which are used in the interviews conducted in this study, are shown in Table 7.

## 6. Experiments and Evaluations of the Proposed System

To test the proposed “mind calligraphy system,” two experiments were conducted in this study. In the first experiment which was conducted in the exhibition of the prototype system, as mentioned in Section 3.1, the observational and interview methods were adopted to collect opinions based on which the prototype system was modified. The second experiment was a formal survey conducted during a formal public exhibition of an improved version of the system also mentioned in Section 3.1, in which the observational, questionnaire-survey, and interview methods were adopted. The details are described in this section.

### 6.1. Analysis of the Results of the First Experiment

In the first experiment, 40 participants who were students, teachers, or staff members in the authors’ university used the proposed system, and they were all 18 years old or over. No limit was put on the participant’s physical status; a disabled person was also welcome as long as he/she had a healthy mental state and normal hands. Furthermore, 25 of the participants were randomly selected to accept the interviews conducted in this study, and every one of them took the interview activity seriously and completed the interview process.

In addition, it was the second author of this paper who conducted the interviews and distributed of the questionnaires to the participants. Additionally, an information consent form was given to each participant for him/her to fill in anonymously (for the case of interviews) or to read (for the case of questionnaire surveys) if he/she was willing to join the activity. During the interview process, the participant had the right to stop the activity and leave anytime, as informed by the second author. Finally, when the participant was filling in the questionnaire, the researchers of this study always kept away to allow the participant to fill in the questionnaire freely with no pressure.

#### 6.1.1. Results of Observations in the First Experiment

During the first experiment, video and text recordings of the entire calligraphy process conducted by the invited participants as writers were carried out. Based on the observation data collected from the video and text records which are described in Table 8, the following conclusions can be drawn.

(1) The writers were not familiar with the usage of the brain wave headset, and sometimes the brain wave signals were not detected due to individual factors.
(2) If the explanation of the operations of the system was insufficient, the writer could not understand the interaction procedure clearly.
(3) Most of the writers understood the way of operating the buttons on the touch screen as the interface with the system.
(4) The interaction pool can attract the attention of the by-standing people.
(5) The writers had positive feelings on the interactive feedback that was produced according to the mental state signals yielded by the brain wave machine.
(6) The writers were willing to discuss and share their emotions that were expressed while they were using the system.

#### 6.1.2. Results of the Interviews of the First Experiment

Twenty-five participating writers who attended the first experiment conducted on the prototype system were selected randomly for interviews in the first experiment as mentioned previously. The indicators of the interview, as shown previously in Table 7, includes three parts, namely, “operation of the man–machine interfacing,” “opinion on the calligraphy,” and ‘opinion on the affective computing.” The writers’ opinions on the questions about these indicators were recorded systematically during the interviews, and are shown in Table 9. The viewpoints extracted from the opinions in the table are summarized as follows.

(1) The writers were curious about the way their emotions were measured by the brain wave machine.
(2) Reminders and guidance of the operational procedure should be enhanced to improve the writer’s performance with the system in the calligraphy process.
(3) Compared with the traditional calligraphy, this system brought about quite different user experiences.
(4) Compared with the traditional calligraphy, the use of this system was richer and more interesting, and can enhance the exchanges of feelings among the writers.
(5) The interactive feedback of this system can help the writer feel their own emotional changes.

### 6.2. Analysis of Data Collected in the Second Experiment

**Figure 8:**
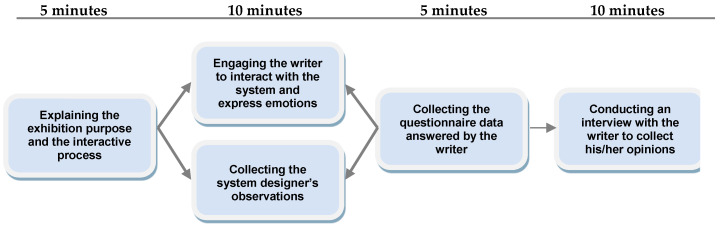
Procedure of the activities conducted in the public exhibition of the proposed system.

The activities involved in the public exhibitions of the system in the second experiment for collecting more opinions to improve the system design are described in more detail in the following and illustrated in Figure 8.

(1) Introducing the system to a participant—by explaining the exhibition’s purpose and the interactive process to a participant who was invited to be a calligrapher using the system.
(2) Engaging the writer in system performance—by assisting the writer to conduct calligraphy and interact with the system to conceal his/her emotions during the writing process.
(3) Collecting the record of the writer’s behavior and the system’s performance—by gathering the system designer’s observations recorded in video or/and paper forms.
(4) Inviting the writer to answer a questionnaire—by asking the writer to fill in a questionnaire about the evaluation of the writer’s behavior and emotions.
(5) Interviewing the writer about his/her experience of using the system—for the purposes of collecting the writer’s opinions about the issues of the man–machine interaction using the system, the calligraphy procedure by the digital approach, and the affective computing process to express the emotions, etc.

The opinions of the writers collected through interviews and the records gathered by the system designer, as described above, were analyzed for improving the system design in this study. More details in this aspect will be presented subsequently. Additionally, in Figure 8, the required time for each activity is shown, and the total time for all the activities was just 30 min, which was not too long, making the entire procedure efficient.

In the second experiment, 57 participants, with their ages and other statuses similar to those of the participants joining the first experiment, used the proposed system, and all of them answered the questionnaire survey, generating 54 valid questionnaires with three being incomplete and so removed. In addition, 25 participants were selected randomly from the 57 to accept the interviews conducted by the researchers of this study.

**Figure 9:**
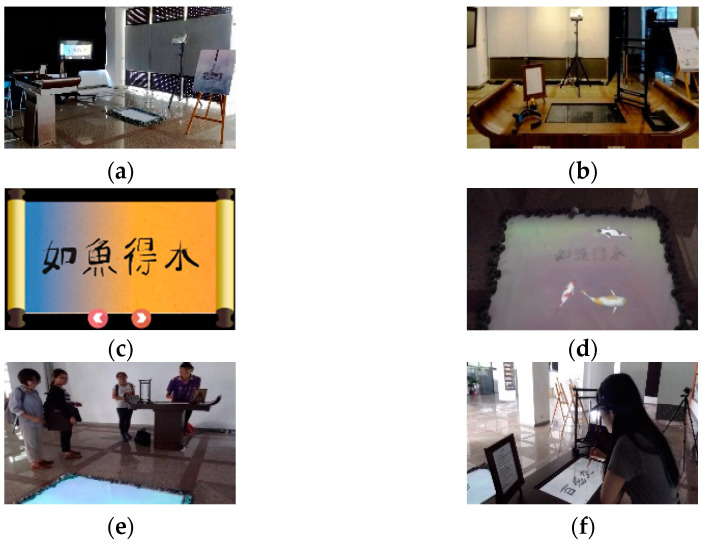
The public exhibition environment of the second experiment. (**a**) The exhibition hall. (**b**) Decoration of the table for calligraphy with the touch screen affixed to the table. (**c**) A completed calligraphic work seen on the display screen. (**d**) A calligraphic work superimposed on an animation shown in the interaction pool. (**e**) Some observers by-standing at the interaction pool. (**f**) A writer conducting calligraphy on the touch screen.

In more detail, it is the second author of this paper who conducted the work of interviewing and distributing the questionnaires to the participants. Additionally, an information consent form was given to each participant for him/her to fill in anonymously (for the case of interviews) or to read (for the case of questionnaire surveys) if he/she was willing to join the activity. During the interview process, the participant has the right to stop the activity and leave anytime, as informed by the second author. Finally, when the participant was filling in the questionnaire, the researchers of this study always kept away to allow the participant to fill in the questionnaire freely with no pressure. Figure 9 shows some images of the environment and activities of the second public exhibition. The data collected from these activities were analyzed, and the results are described in this section.

#### 6.2.1. Results of Observations Collected from the Public Exhibition

Based on the observation data collected from the public exhibition of the second experiment, which are described in Table 10, the following facts can be drawn.

(1) The writers were not familiar with how to wear and use the brain wave machine, which was a headset.
(2) After reading the operation instructions, the writers could use the proposed system by themselves.
(3) The feedback of the touch pen on the touch screen was slow.
(4) The interaction pool and the display screen could attract the attention of the writer.
(5) When writing, the writer could receive the real-time feedback of the system via hearing the play of the sound in the animation.
(6) The writers were willing to discuss and share the changes of their emotions and calligraphic works.

#### 6.2.2. Results of Questionnaire Surveys

A total of 54 valid pieces of feedback from the participating writers were collected in the questionnaire survey of the second experiment; the questionnaire included questions about the participant’s basic data, and the evaluations of the two indicators of system usability and writer’s experience. The questions of the two indicators are shown in Table 6. Some statistics of the collected feedback data of the Likert 5-point scale of the two indicators are listed in Table 11 and Table 12. They were analyzed in detail from several points of view, as described in the following.

Note that since questions B2, B5, and B9 are negative in wording, in order to maintain the consistency of the average scores, the scores and the percentages of “agree” and “strongly agree” of these questions have been interpreted as “disagree” and “strongly disagree,” respectively, and vice versa, while computing the data in Table 11 and Table 12.

(1) Analyzing the basic data of the participants in the exhibition

From the results of the collected basic data about the participants who attended the exhibition, 72.2% of them mentioned that they had learned calligraphy before, while 96.2% of the participants had used the proposed interactive system.

(2) Designing the processes for testing the various properties of the collected questionnaire data

In this study, the SPSS and AMOS software packages were used to analyze the collected questionnaire data, while the Microsoft Excel application program was used for tabulation of the results. Specifically, a series of tests were conducted to verify the various properties of the collected data to ensure that the data can be analyzed for their usefulness for the calligraphy application. The data properties and the methods adopted to verify them are listed in the following, with the details described later in this section and in Appendix B.

1. Adequacy of the collected data—verified by the Kaiser–Meyer–Olkin (KMO) test and Bartlett’s test of sphericity using the SPSS package.
2. Latent dimensions (scales) of the questions used in collecting the data—found by exploratory factor analysis (EFA) via the principal component analysis (PCA) method and the varimax method with Kaiser normalization using the SPSS package.
3. Reliability of the collected data—verified by use of the Cronbach’s α coefficient values yielded by the EFA process.
4. Suitability of the model structure of the data set up according to the found question dimensions (scales)—verified by confirmatory factor analysis (CFA) using the AMOS package.
5. Validity of the collected questionnaire data—verified by parameter values yielded by the EFA and CFA processes.

(3) Testing the adequacy of the collected questionnaire data

To evaluate the adequacy of the data collected through the questionnaire survey, the Kaiser–Meyer–Olkin (KMO) test and the Bartlett’s test of sphericity were adopted in this study [42,43,44,45,46,47]. The KMO measure is a statistic used to indicate the proportion of variance among the variables that might be caused by certain factors underlying the variables. The KMO test returns measure values between 0 and 1, and Kaiser [42] classified the returned values as follows: (1) 0.00 to 0.49—unacceptable; (2) 0.50 to 0.59—miserable; (3) 0.60 to 0.69—mediocre; (4) 0.70 to 0.79—middling; (5) 0.80 to 0.89—meritorious; and (6) 0.90 to 1.00—marvelous. A KMO measure value larger than the threshold value 0.50 is usually taken to be acceptable to pass the test [44,45]. Additionally, the Bartlett’s test of sphericity is used to test the hypothesis that the correlation matrix of the data variables is an identity matrix, indicating that the variables are unrelated. A significance value of the test result smaller than the threshold value 0.05 is usually taken to be acceptable to reject the hypothesis, or equivalently, to pass the test [45,46]. When both of the two tests are passed, the data variables are usually said to be adequately related for further structure analysis [47].

Using the collected questionnaire data and their statistics shown in Table 11 and Table 12, the KMO measure values and the significance values of the Bartlett’s test for the two indicators computed by the SPSS are listed in Table 13, which is a summary of the results shown in Appendix B.1 in Appendix B. From the table, it can be seen that for either of the two indicators, the KMO measure value is larger than the threshold of 0.5 and the significance value of the Bartlett test is smaller than the threshold 0.05; therefore, both data sets of the two indicators are adequately related for further structure analysis, as described next.

(4) Finding the latent question dimensions (scales) of the questions from the collected data

With the adequacy of the questionnaire data being verified, the SPSS was used to perform exploratory factor analysis (EFA) by principal component analysis and the method of varimax with Kaiser normalization to find suitable latent dimensions (scales) for the questions with the collected data as inputs. The details can be found in Appendix B.2 in Appendix B, from which it can be seen that the 12 questions of the first indicator, system usability, can be divided into three groups under the question dimensions (scales) of ease to learn, efficiency, and satisfaction, respectively; and the 10 questions of the second indicator, writer’s experience, can be divided into three groups as well under the question dimensions (scales) of affective or cognitive experience, sensory experience, and social-identity experience or behavior, respectively. Table 14 shows integrally the results of such latent dimension (scale) findings with some statistics of the Liker’s scale data included.

(5) Verifying the Reliability of the Collected Data by the Cronbach’s α Coefficients

Reliability is about the consistency of a measured data set despite of the repeated times [48]. In this study, the Cronbach’s α coefficient [49,50] yielded by the EFA mentioned previously was adopted in this study to analyze the reliability of the questionnaire data. It is known that the closer the Cronbach’s α coefficient of a data set of a scale is to the extreme value 1.0, the greater the reliability of the data set (regarded as variables) is. According to Gilford [51], the following rules can be used to judge the goodness of the reliability of a data set:

$$\begin{array}{lll} \alpha \geq 0.70 & \to & \mathrm{highly}\;\mathrm{reliable};\\ 0.35\leq \alpha <0.70 & \to & \mathrm{reliable};\\ \alpha \leq 0.35 & \to & \mathrm{unreliable}, \end{array}$$

where α is the Cronbach’s α coefficient value.

According to Table A7 and Table A8 in Appendix B.3 in Appendix B, the Cronbach’s α coefficient values of the six question dimensions (scales) and those of the two indicators are shown integrally in Table 15. It can be seen from the table that all the Cronbach’s α coefficient values are in the range of 0.35 to 0.70 or even larger, meaning that the collected questionnaire data of each question dimension and those of each indicator are reliable.

(6) Verifying the suitability of the structure model set up by the question dimensions (scales)

**Figure 10:**
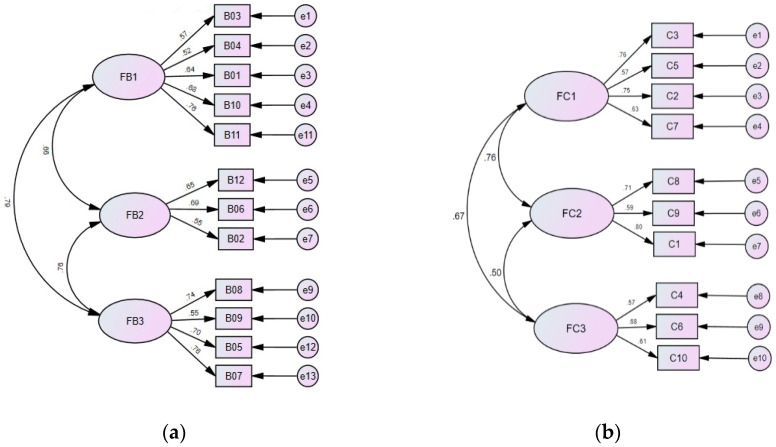
The 3-scale structure model graphs yielded by the CFA of the two indicators of “system usability” and “writer’s experience.” (**a**) Graph of the indicator of “system usability” (FB1: ease to learn; FB2: efficiency; FB3: satisfaction). (**b**) Graph of the indicator of “writer’s experience” (FC1: affective or cognitive experience; FC2: sensory experience; FC3: social-identity experience or behavior).

Before proving the validity of the collected questionnaire data, the suitability of the structure model set up by the question dimensions (scales) must be verified [52]. For that aim, the confirmatory factor analysis (CFA) process using the AMOS package was applied on the collected questionnaire data, as described in detail in Appendix B.4 in Appendix B, yielding two 3-scale structure-model graphs as shown in Figure 10. Furthermore, a list of structure-model fit indices was yielded by the CFA for each indicator, including the degrees of freedom (df), the chi-square (χ^2^) statistics, the ratio of χ^2^/df, the adjusted goodness-of-fit index (gfi), the comparative fit index (cfi), and the root mean square error of approximation (RMSEA), as shown integrally in Table 16. According to the analyses made in Appendix B.4 in Appendix B, the index values of χ^2^/df, gfi, cfi, and RMSEA yielded for each indicator the fact that the structure model set up by the question dimensions (scales) of the indicator is of a reasonably good fit to the collected questionnaire data [53,54,55,56,57].

(7) Proving the validity of the collected questionnaire data

With the model structures of the two indicators both proved to fit reasonably to the collected questionnaire data, it is appropriate to analyze further the validity of the data. It can be seen from the 3-scale structure model shown in Figure 10 that all the factor loading values (also called standardized regression weights) with respective to the scales (appearing on the paths from the scales FB1–FB3 and FC1–FC3 to the questions B01–B12 and C1–C10, respectively) are all larger than the threshold of 0.5, meaning that the construct validity of the model was verified; and this fact can also be proved by the construct validity values of all the scales of the two indicators of system usability and writer’s experience yielded by the EFA process mentioned above, which are listed in Table 17 and can be seen to all be larger than the threshold value of 0.6 [58,59]. That is, the construct validity of the collected questionnaire data of the indicator of system usability is proven.

(8) Summary of questionnaire surveys

It can be concluded from the above discussions that the questionnaire data collected from the writers about the two indicators of system usability and writer’s experience are both reliable and valid for uses in further analyses of the data contents, which lead to the following conclusions.

(A) Evaluation of the system usability:

About the evaluation of the usability of the proposed system, the overall feedback of the questionnaire survey was positive, as can be seen from the upper part of the last column of Table 11 where the average percentage of agreement is seen to be 85.7%, which says that the writers generally think that the usability of the system is good. Among the 12 questions, questions B1, B3, B4, B7, and B11, which are about the performance of the system, had scores higher than the average value of 4.15, indicating more than 90% of the participants recognized the good performance of this system, as can be seen from the upper parts of Table 11 and Table 12. Two questions with lower scores were B2 and B12.

The above analysis about the system usability leads to the following conclusions.

(a) The writers believed that the proposed system was easy to use, so the design of the operation interface was good.
(b) The writers felt happy to use the system and interested in the interaction, so they were satisfied with the way of presentations of the proposed system.
(c) Some writers watched the interaction pool when they wrote, and they were also distracted by the display screen at the remote site.
(d) The feedback of writing on the touch screen was slow, so the sensitivity and fluency of the touch screen need be improved.

(B) Evaluation of the writers’ experiences:

About the evaluation of a writer’s experience of using the system, most questions had positive feedback, as can be seen from the lower part of the last column of Table 11, and the average percentage of agreement was seen to be 81.7%, which says that the overall experience obtained by the writer was good. Among the 10 questions, the average scores of four questions (C1, C5, C8, and C10) were over 4.2, as can be seen from the lower part of Table 12, and the corresponding proportions of agreements were more than 90%, as can be seen from Table 11, indicating that the experiences of interaction with the system obtained by the writers were good. Two questions with lower scores were C2 and C7. More detailed analyses about the writers’ experiences of using the system led to the following conclusions.

(a) The writers had positive feelings for the interactive technology which enhances calligraphy; through this system, they experienced calligraphy in a new way.
(b) By analysis of the writers’ emotions via the brain wave machine, the feedback of the system let them understand their emotional changes.
(c) It was difficult for the writer to understand the way to control emotions during the system experiencing process because the system is designed to focus on the emotional visualization and feedback with no direct instruction for the writer to control his/her emotion.
(d) Besides emotions, there were many meanings of calligraphy; however, the feedback of emotions was the main purpose of this system, so it was difficult for the writer to fully understand the meanings of calligraphy through this system.
(e) The writer was highly positive of the method that combined interactive technology with traditional calligraphy.

(9) Evaluations of the system usability and the writer’s experience from the question-dimension viewpoints.

As for the evaluation of the system usability from the viewpoints of the three question dimensions of “ease to learn,” “efficiency,” and “satisfaction,” the average scores of “ease to learn” and “satisfaction” were larger than four and the proportions of agreement (“strongly agree” plus “agree”) were larger than 90%, as shown in Table 18 and Table 19, whose data were computed according to those shown in the upper parts of Table 11 and Table 12. These results indicate that the writer can have good performances while using the proposed system. As of “efficiency,” the average score 3.83 was smaller than four, and the proportion of agreement was 72.84% while the proportion of disagreement was 9.27%, which shows that the proposed system should be improved in this aspect.

About the evaluation of the writers’ experiences from the viewpoints of the three question dimensions of “affective or cognitive experience,” “sensory experience,” and “social-identity experience or behavior,” all scores were larger than four, as can be seen from Table 20 and Table 21, whose data were computed from the lower parts of Table 9 and Table 10. These results indicate that most of the writers’ experiences were good.

(10) Conclusions of the evaluations of questionnaire data contents

The results of the questionnaire survey, as discussed above, show that the questionnaire data have good reliability and validity. In the questionnaire, the average scores of the answers to 80% of the questions were larger than four, which indicates that the writers gave positive evaluations of the proposed system. Specifically, about the evaluation of the system usability, the positive attitude of the writers shows that the system is easy to operate and that they were satisfied with the interactive process. However, it will be better if the precision of writing using the system can be improved. As to the survey results of the writers’ experiences, the displays and feedback of this system were the most attractive to them. It is also good for the writer to express calligraphy through the affective computing technology provided by the proposed system.

In more detail, the results of the questionnaire survey are summarized as follows.

(a) The writers felt that the operation interface of the proposed system was easy to learn and use.
(b) The writers were satisfied with the combination of the interactive technology and the affective computing technology, and thought the outcome to be interesting.
(c) The sensitivity of the touch screen should be improved in order to make the writing process more fluent.
(d) The writers learned how calligraphy interacted with their own emotions via the feedback of the system in the interactive process, and understood further the relationship between calligraphy and emotions.
(e) The proposed system increases the willingness of the writers to be exposed to calligraphy through interactive devices.

### 6.3. Summary of Results of Interviews with the Writers Attending the System Exhibition

Twenty-five writers who attended the second public exhibition of the system were selected randomly for interviews, as mentioned. For each interview question, if more than 75% of the writers had similar opinions, they were regarded as the majority; and if certain opinions were expressed by only one or two writers, they were regarded as the minority. The questions asked in the interviews included three indicators, namely, “man–machine interface,” “opinion on calligraphy,” and “opinion on affective computing.” The opinions of the writers were systematically recorded, as shown in Table 22.

The results of the interviews are summarized below.

(1) The means of interaction with the brain wave machine was positively evaluated by the writers.
(2) The fluency of the touch screen was better than that of the first experiment, but it still can be improved.
(3) The system provided the writers with novel and rich interactive experiences of calligraphy, which can be applied to children and beginners as well.
(4) The system allowed the writers to recall the memories of calligraphy and enhanced their willingness to contact calligraphy once again.
(5) The interactive calligraphy process of the proposed system can enhance the writers’ willingness to experience calligraphy.
(6) Through interactions with the proposed system, the writers can understand the relation between calligraphy and emotional expressions.
(7) The proposed system provided calligraphy experiences that are different from those provided by the traditional calligraphy approach.

### 6.4. Summary of Research Findings

During the first experiment, the proposed system in its prototype form was evaluated through the observational method and the interview method. After being improved according to the evaluation results, the system was formally exhibited in the second experiment. The results obtained in the formal exhibition were analyzed by the observational, questionnaire-survey, and interview methods. After a series of cross-analyses of the various evaluation results described in the last section, the main findings obtained in this study can be summarized as follows, which are focused more on the writer’s affections using the proposed “mind calligraphy” system.

(1) The interactive system can raise the writer’s interest in engaging in calligraphy while interacting with the system.
(2) The usage of the system can reveal the writer’s emotions regarding focusing, relaxation, calmness, and anxiety during calligraphy through the animations of fish swimming, and sounds of raindrops, insects, and thunder played on the system.
(3) Visualizing the writer’s emotions through animations enhances the writer’s cognitive engagement in the calligraphy.
(4) Real-time feedback of the system increases the writer’s willingness to continue the calligraphy process.
(5) The engagement of the writer in the system’s performance with various forms of interactions provides the writer with a new experience in calligraphy.

In view of the main purpose of promoting “mind calligraphy” by affective computing and visualization techniques, an overall concluding remark made in this study according to the above evaluations is that the goal has been accomplished.

## 7. Conclusions

In this study, the affective computing and visualization techniques have been used to construct an interactive system for performing “mind calligraphy,” aiming at helping a calligrapher to understand the relationship between calligraphy and emotions in a simpler way and to realize that emotion is also one of the values of calligraphy. The ideas for designing a prototype system were based on conclusions drawn from literature reviews and expert interviews. A real interactive calligraphy system was developed through improvements on the prototype system which were based on opinions collected from three types of activities conducted in public exhibitions, namely, observations, a questionnaire, and interviews. For the purpose of implementing the proposed “mind calligraphy” system, a brain wave machine was used to measure the writer’s mental states continuously, which were classified to into four types of emotion. Multimedia feedback of animations, sounds, and color palettes corresponding to the emotional type was then generated as the response and displayed for inspection by the writer and by-standing observers. In this way, a new form of calligraphy experience was brought about to the writer, and may be said to redefine the calligraphy art by a digital approach. By cross-analyses of the opinions collected from the public exhibitions, the following six conclusions were reached.

(1) Human emotion is an important expression in calligraphy

The literature surveys conducted in this study help us to understand the fact that the writer’s emotion affects his/her writing of character strokes. Although many people thought calligraphy is just to write the words well, calligraphy experts also pointed out that what is more important is the mood in writing. Writing in the context of emotional turmoil allows the writer to inject particularity into the works, possibly so as to create a personal calligraphy style.

(2) The integration of affective computing and visualization techniques into calligraphy creates a new innovative calligraphy art.

Though creative ideas have been proposed by some existing studies to allow a writer to experience calligraphy more deeply and artistically, few methods have been proposed for exploring the important issue of emotional expressions in calligraphy. In addition, most of these methods did not let the writer actually write. In this study, affective computing and visualization techniques were combined with calligraphy to allow the participating writer to obtain a new and innovative calligraphy experience.

(3) The way of interaction with the brain wave machine used in the calligraphy system offers a new human–machine interactive experience.

The use of the brain wave machine in testing concentration and relaxation is very extensive nowadays. It coincides with the purpose of calligraphy sought in this study for a writer to pursue calmness and concentration through the writing process. During the exhibitions of the proposed system, it was found that most of the writers had never used the brain wave machine before. Although they did not understand the modes of operation of the device, after their interactions with the system, they all had a high degree of interest and acceptance of the new method of brain wave-based interaction in the affective computing system.

(4) The visualization of the invisible emotion helps the writer understand the relationship between calligraphy and his/her emotions.

Emotions accompany human beings all the time; if not properly expressed, they will affect people inherently in their daily lives now and then. In the domain of calligraphy, experts explore the writer’s mental state through stroke writing. However, it is difficult for the general public to understand this principle. Therefore, it is meaningful for the proposed system to use the brain wave machine to read the mental state signals and transform them into audio-visual forms (animations, sounds, color palettes) for inspection, allowing the writer to have a more intuitive understanding of the relation between calligraphy actions and emotion expressions.

(5) The interaction mechanism provided by the proposed system can be used to interpret calligraphy, change the audience’s views of this art, and enhance people’s willingness to try calligraphy.

The participating writers expressed in the interviews that the way of experiencing calligraphy interactively through the use of the proposed system changed their opinions on calligraphy. They used to believe that calligraphy can mainly be adopted for writing; after experiencing the interaction with the proposed system, they believed that calligraphy can be practiced in a relaxed and pleasant manner. Additionally, to use the proposed interactive system, no preparation of writing props is required, meaning that the system is environmentally friendly. Furthermore, the interaction of the system was presented in a way that not only attracts the public’s attention to the calligraphy, but also enhances the writer’s ideas and willingness to experience calligraphy.

(6) The timely feedback offered by the system allows the writer to share opinions quickly with others, increasing the degree of enjoyment and pleasure of viewing exchanges about calligraphy

The system reflects the writer’s emotions in real time via the animation shown on the panel of the interaction pool, which makes the writer and the by-standing observers feel calligraphy is interesting. Additionally, after a calligraphic work is completed, it can be superimposed on an emotion-representing color palette and sent to the display screen to show to other bystanders. This interactive presentation allows the writer to attract the attentions of the by-standers and enhance his/her willingness to exchange viewpoints about calligraphy with them.

It is hoped in this study that the general public can realize the important emotional aspects of calligraphy through the techniques of affective computing and visualization implemented upon the proposed system, and understand the fact that calligraphy is not only a practice of stroke writing, but also an art of emotion expression. It is expected in addition that by combining the uses of human–computer interfacing and affective computing techniques, people’s impressions of traditional calligraphy can be changed. It is desired as well that the art of calligraphy can be “valued” to a greater degree in the digital age, and that its value and implications can be extended continuously by means of the proposed system.

Finally, though the system was designed for the purpose of calligraphy, the way of employing affective computing and visualization techniques and deploying interactive devices to construct the proposed “mind calligraphy” writing system can be emulated in other fields, such as painting, article composition, game playing, etc., to reveal the participant’s mental state or emotions in real time for various application purposes. Furthermore, the sample sizes of the participants in the experiments conducted in this study were not large; larger samples with sizes up to 250 should be considered in future studies for the results of system evaluations to be more convincing.

## Figures and Tables

**Table 1:** Classification of brain wave signals into four types of emotions with representative colors.

Emotion Type	Classification Rules *	Representative Color
Focusing	*a* ≥ 70 & *a* ≥ *m*	Blue
Relaxation	*m* ≥ 70 & *m* > *a*	Green
Calmness	30 ≤ *a* < 70 & 30 ≤ *m* < 70	Orange
Anxiety	*a* < 30 or *m* < 30	Aubergine

* Note: ‘*a*’ and ‘*m*’ mean attention and meditation, respectively.

**Table 2:** Descriptions of emotions and corresponding color palettes.

Color Weights of Emotion	Color Palette	Description
Focusing: 0; Relaxation: 2; Calmness: 1; Anxiety: 1	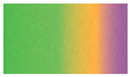	The number of occurrences of relaxation are 2, while those of anxiety and calmness are both 1.
Focusing: 0; Relaxation: 2; Calmness: 0; Anxiety: 2	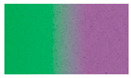	The number of occurrences of relaxation are 2, while those of anxiety are 2.
Focusing: 1; Relaxation: 1; Calmness: 1; Anxiety: 1	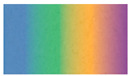	The number of occurrences of focusing, relaxation, calmness, and anxiety are the same.
Focusing: 3; Relaxation: 0; Calmness: 0; Anxiety: 1	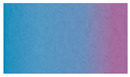	The number of occurrences of focusing are 3, while that of anxiety is 1.
Focusing: 1; Relaxation: 1; Calmness: 2; Anxiety: 0	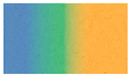	The number of occurrences of focusing and relaxation are both 1, while those of calmness are 2.
Focusing: 2; Relaxation: 2; Calmness: 0; Anxiety: 0	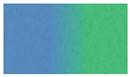	The number of occurrences of focusing are 2, while those of relaxation are 2.

**Table 3:** List of the animation scenarios played on the panel of the interaction pool.

	Emotion	Focusing	Relaxation	Calmness	Anxiety
Effect					
Color shown on pool background		Blue	Green	Orange	Aubergine
Number of raindrops		A small number	A small number	A large number	A huge number
Direction & speed of fish movement		In the same direction with a medium speed	Up and down with a slow speed	In different directions with a medium speed	In chaotic directions with a fast speed
Sound type of played audio		Raindrop	Insect	River	Thunder
Descriptions of animations and corresponding meanings		(1) Fishes swim in the same direction with a medium speed, meaning that the writer is focusing on writing. (2) A small number of raindrops fall, meaning that the writer is paying a certain degree of attention to writing. (3) The raindrops sound clear, meaning that the writer seems to have a very strong intention and his/her mind is concentrated.	(1) Fishes swim at the same place to move up and down slowly, and a small number of raindrops fall, meaning that the writer is in an easy mood during the writing. (2) Insect sounds can be heard, meaning that the writer feels relaxed.	(1) Fishes roam freely in different directions with a medium speed, and many raindrops fall slightly, meaning that the writer is in calmness during writing. (2) The sound of a river can be heard, meaning that the writer’s mind is calm.	(1) Fishes swim fast in chaotic directions with fast speeds, and lots of raindrops fall with noise, meaning that the writer is anxious in writing. (2) The sounds of thunder are heard, meaning that the writer feels anxious.

**Table 4:** List of intermediate interaction results of performing Algorithm 3.

No.	Action	Corresponding Step in Algorithm 3	Involved Interaction Device	Illustration of Intermediate Result
1	The system is in the standby state (with an initial animation played on the interaction pool).	None	Interaction pool	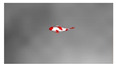
2	The writer wears the brain wave headset.	None	Brain wave machine (headset)	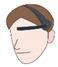
3	The writer clicks the button of "select" to choose a character set.	Step 1	Touch screen	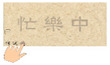
4	The writer uses the pen brush to conduct calligraphy.	Step 2.1	Touch screen	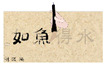
5	The system plays the animation of the emotion type decided from the brain wave.	Step 2.2(a)	Interaction pool	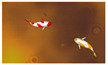
6	The writer pushes the button of ‘send’ to send the calligraphic work to the interaction pool.	Step 2.2(b)	Touch screen	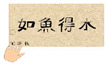
7	The writer’s work is shown in a floating manner in the animation in the interaction pool.	Step 2.2(b)	Interaction pool	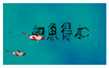
8	To end a writing session, the writer uses the stamp to create a seal shape, and send the result to the server to keep.	Step 2.2(c)(d)	Touch screen	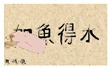
9	The result above is shown on the touch screen with a color palette as the background.	Step 2.2(d)	Touch screen	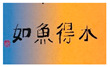
10	The result above with the color palette background is sent to the display screen to show to the observer there, who can use the advance icon to change the displayed work.	Step 2.2(d)	Display screen	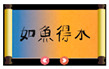

**Table 5:** List of items for observations of the two indicators of system usability and writer’s behavior.

Indicator for Observation	Observed Item
System usability	Can the writer understand the way of interaction with the system?
	Can the writer wear the brain wave instrument properly?
	Can the writer perform the system properly?
Writer’s behavior	Is the writer interested in the system and use it?
	What are the responses of the writer when he/she interacts with the system?

**Table 6:** List of questions of the questionnaire for evaluating system usability and writers’ experiences.

Indicator	Lebel	Question
System usability	B1	I think it is easy to operate this system.
	B2	I cannot concentrate to use this system.
	B3	I can quickly perform each step of using the system.
	B4	I can operate the interface of this system.
	B5	This system fails to attract my attention.
	B6	I think this system has effectively integrated several functions.
	B7	I like the way of presentations of this system.
	B8	I think the feedback of this system is rich.
	B9	I cannot utilize each item of the system.
	B10	I can clearly understand the feedback of this system.
	B11	I feel the interaction of the system interesting.
	B12	I can use this system smoothly.
Writer’s experience	C1	The way of operating the system can attract my attention.
	C2	This system can help me control emotions better.
	C3	I am happy after using the interactive device of this system.
	C4	This system changes my opinion on calligraphy.
	C5	The audio-visual feedback of the system can attract my attention.
	C6	This system can help me understand better the meaning of calligraphy.
	C7	I am more willing to know more about calligraphy after using the interactive system.
	C8	I can feel the change of emotions from the feedback of the system after using the interactive device of this system.
	C9	This system makes me aware of the importance of emotion expressions.
	C10	I am more willing to experience interactive and scientific works after using the device of the interactive system.

**Table 7:** List of the questions used in the interviews with the writers using the proposed system.

Indicator of the Interview	Question of the Interview
Operation of the man--machine interfacing mechanism	What do you think of the interaction based on the use of the brain wave?
	Do you feel it easy to use the touch screen to conduct calligraphy?
	What do you think of the interfacing operations of this system?
Opinion on the calligraphy procedure	What is the difference between the current digital approach and the traditional approach to calligraphy?
	Can this interactive system enhance your willingness to know more about calligraphy?
Opinion on the affective computing process	Can you feel your emotional change based on the feedback of the system?
	Can you feel that emotions are related to the calligraphy after you interacted with the system?

**Table 8:** Observation results collected from the public exhibition of the first experiment.

Indicator for Observation	Item for Observation	Observation Result
Operation conditions of the system	Whether the writer can understand the way of interaction with the proposed system.	The writer knew how to use the system after listening to an introduction to the system. Receiving no explanation about the system operations, the writer did not know how to conduct the interaction process.
	Whether the writer can wear the brain wave instrument properly.	Most writers need the researcher’s help to put on the brain wave machine. Some writers were worried about breaking the machine.
Writer’s behavior	Whether the writer can operate the proposed system properly.	Sometimes, the brain wave machine fails to collect data due to the writer’s head size and hair style. Due to insufficient sensitivity, the pen brush sometimes cannot be used to write smoothly on the touch screen. The writer does not know how much water the pen brush should dip for smooth writing. After receiving an introduction to the system process, the writer can understand the functions of the user interface quickly and control it properly. Some writers misunderstood the file-saving function as being identical to the seal-stamping function.
	Whether the writer is interested in the devices of the proposed system and use them.	Most writers were attracted by the projected animation in the interaction pool. Being attracted by the work appearing on the display screen, some writers would try to create similar works by themselves.
	What the responses of the writer are when he/she interacts with the system.	The writers misunderstood that all the pen brushes on the brush holder can be used. Until finishing the writing tasks, most writers concentrated on writing by touching the touch screen without noticing the changes in the interaction pool. The writers responded more to the audio feedback. Bystanders paid more attention to the animation appearing in the interaction pool. The writer would wait for the feedback to appear on the touch screen after seal stamping. The writer would wait for the appearance of his/her own writing to appear on the display screen. The writer would discuss and share the feedback incurred by the brain wave change with bystanders.

**Table 9:** Interview results obtained from the writers participating in the first experiment.

Indicators	Question of Interview	Record of Interview Opinion
Operation on man-machine interfacing	What do you think of the interaction based on the use of the brain wave?	Most writers thought the interaction with the brain wave machine novel and interesting. (17) * The writers thought that the brain wave machine could be used to inspect his/her own feeling in his/her mind. (5) The writer would get confused when he/she did not know how to control his/her brain wave. (3)
	What do you think of write calligraphy on a touch screen?	Some writers thought that the sensitivity of the touch screen for writing should be improved. (6) Some writers thought that the experience of writing on the touch screen and that of the brush writing of traditional calligraphy are different. (8) Some writers thought writing via the touch screen is novel and interesting. (8) The writers thought that writing on the touch screen was more convenient and environment-friendly. (3)
	What do you think of the operation interface of this system?	The writers thought the entire operation interface is simple. (12) The writers indicated that the sensitivity of the touch screen will affect their operations. (5) Some writers hoped that more guidance of seal stamping can be given. (4) Some writers did not know how to return to the touch screen for a new phase of writing. (3) A few writers hoped that the system could have the function of allowing arbitrary writing. (1)
Opinions on calligraphy	What do you think of the digital technique of expression of calligraphy, compared with the traditional calligraphy?	Many writers thought that the proposed way of calligraphic writing is more interesting and richer than traditional calligraphy, arousing many feelings of the writers. (12) Some writers thought that this system allowed them to share their own works with others (4) Some writers thought that the digital way of calligraphy decreases the time to prepare the tools for calligraphic writing. (4) Some writers thought the digital way of calligraphy lost the traditional pen brush touch in writing, reducing the realism of the pen brush writing. (4) A very few writers thought that the digital way of calligraphy is not so different from the traditional calligraphy. (1)
	Can this interactive system enhance your willingness to know more about calligraphy?	Most writers thought that this system increased their willingness to engage in calligraphy. (17) Some writers thought that this system did not increase their willingness to re-engage in calligraphy. (8)
Opinions on affective computing	Can you feel your emotional change based on the feedback of the system?	Most writers could feel their emotions through the system feedback (sound playing and animation changes in the interaction pool). (20) Some writers mentioned that they could not notice the visual changes in the interaction pool when writing because of their concentration on writing. (5)
	Can you feel that emotions are related to calligraphy after you interacted with the system?	Most writers mentioned that in the process of interaction with the system, they could feel the interrelation between their emotions and writing actions. (22) Some writers pointed out that there was no correlation between their emotions and writing actions in the experiencing process. (3)

* Note: the number in parentheses means the number of writers giving the opinions.

**Table 10:** Observation results collected from the public exhibition of the second experiment.

Indicator for Observation	Item for Observation	Observation Result
Operation conditions of the system	Whether the writer can understand the way of interaction with the proposed system.	After listening to the explanation, the writers immediately knew that this system was an interactive design for Chinese calligraphy and emotion detection.
	Whether the writer can wear the brain wave instrument properly.	Most of the writers needed the researcher’s help to put on the brain wave machine.
The writer’s behavior	Whether the writer can operate the proposed system properly.	Sometimes, the brain wave machine failed to collect data due to the writer’s head size and hair style. The feedback speed of the pen brush on the touch screen was slow which affected some writers. After reading the operation procedure, the writer can quickly understand the functions of the user interface and control it by himself/herself. Some writers did not know that there was an order between “send the calligraphy result” and “stamp the seal” (the latter should be done first).
	Whether the writer is interested in the devices of the proposed system and use them.	Most writers were attracted by the projected animation in the interaction pool. Some writers were attracted by the display screen and operated it.
	What the responses of the writer are when he/she interacts with the system.	While writing, most of the writers concentrated on the writing operation applied on the touch screen and paid less attention to the animation appear on the interaction pool. But they would look at the animation after writing. The writer who participated in the interaction alone tended to have the state of attention. The audio feedback allowed the writer to know his/her emotional state at the moment of writing. The writer, when did not write, paid more attention to the animation appearing on the interaction pool. The writer would stand in front of the display screen to look at the calligraphic works of theirs and others, and discussed the working details with one another. The writers were willing to share and discuss with others about the feedback produced by the changes of the brain wave signals.

**Table 11:** Score percentages of the collected data of the two indicators of system usability and writer’s experience.

No.	Strongly Agree (5 Scores) (A)	Agree (4 Scores) (B)	No Opinion (3 Scores) (C)	Disagree (2 Scores) (D)	Strongly Disagree (1 Scores) (E)	Percentage of Agreements (F = A+B)
B1	48.1	48.1	3.8	0.0	0	96.2
B2	16.7	53.7	18.5	11.1	0	70.4
B3	48.2	42.6	7.4	1.8	0	90.8
B4	42.6	53.7	3.7	0.0	0	96.3
B5	25.9	57.4	12.9	3.8	0	83.3
B6	25.9	57.4	11.1	5.6	0	83.3
B7	46.3	50.0	3.7	0.0	0	96.7
B8	25.9	57.4	13.0	3.7	0	83.3
B9	29.6	55.6	13.0	1.8	0	85.2
B10	31.4	55.6	13.0	0.0	0	87.0
B11	38.9	51.9	9.2	0.0	0	90.8
B12	18.5	46.3	24.1	11.1	0	64.8
**Average**						**85.7**
C1	38.9	55.5	5.6	0	0	94.4
C2	25.9	44.4	20.4	9.3	0	70.3
C3	33.3	48.1	14.8	3.8	0	81.4
C4	33.3	40.8	24.0	1.8	0	74.1
C5	33.3	59.3	5.6	1.8	0	92.6
C6	27.9	48.1	22.2	1.8	0	76.0
C7	13.0	48.1	33.3	5.6	0	61.1
C8	38.9	53.8	3.7	1.8	1.8	92.7
C9	22.2	55.6	22.2	0	0	77.8
C10	48.1	48.1	3.8	0	0	96.7
**Average**						**81.7**

**Table 12:** Statistics of the collected data of the two indicators of system usability and writer’s experience.

Label	Question	Min	Max	Mean	Standard Deviation
B1	I think it is easy to operate this system.	3	5	4.44	0.57
B2	I cannot concentrate to use this system.	2	5	3.75	0.86
B3	I can quickly perform each step of using the system.	2	5	4.37	0.70
B4	I can operate the interface of this system.	3	5	4.39	0.56
B5	This system fails to attract my attention.	2	5	4.01	0.73
B6	I think this system has effectively integrated several functions.	2	5	4.03	0.77
B7	I like the way of presentations of this system.	3	5	4.43	0.56
B8	I think the feedback of this system is rich.	2	5	4.03	0.79
B9	I cannot utilize each item of the system.	2	5	4.13	0.69
B10	I can clearly understand the feedback of this system.	3	5	4.19	0.64
B11	I feel the interaction of the system interesting.	3	5	4.30	0.63
B12	I can use this system smoothly.	2	5	3.72	0.89
**Average**				**4.19**	
C1	The way of operations of the system can attract my attention.	3	5	4.33	0.58
C2	This system can help me control emotions better.	2	5	3.87	0.90
C3	I am happy after using the interactive device of this system.	2	5	4.11	0.79
C4	This system changes my opinion on calligraphy.	2	5	4.06	0.80
C5	The audio-visual feedback of the system can attract my attention.	2	5	4.24	0.64
C6	This system can help me understand better the meaning of calligraphy.	2	5	4.01	0.76
C7	I am more willing to know more about calligraphy after using the interactive system.	2	5	3.68	0.77
C8	I can feel the change of emotions from the feedback of the system after using the interactive device of this system.	1	5	4.26	0.77
C9	This system makes me aware of the importance of emotion expressions.	3	5	4.02	0.69
C10	I am more willing to experience interactive and scientific works after using the device of the interactive system.	3	5	4.45	0.57
	**Average**			**4.10**	

**Table 13:** The KMO measure values and the significance values of the Bartlett’s test of the collected questionnaire data of the two indicators of system usability and writer’s experience.

Indicator	Name of Measure or Test		Value
System usability	KMO measure of sampling adequacy		**0.796**
	Bartlett test of sphericity	Approx. Chi-Square	228.865
		Degree of freedom	66
		Significance	**0.000**
Writer’s experience	KMO measure of sampling adequacy		**0.735**
	Bartlett test of sphericity	Approx. Chi-Square	185.107
		Degree of freedom	45
		Significance	**0.000**

**Table 14:** The question dimensions (scales) of the questions of the two indicators of system usability and writer’ experience by exploratory factor analysis using the SPSS.

Label	Question Dimension	Question	Min	Max	Mean	S.D.
B3	Ease to learn (Group FB1)	I can quickly perform each step of using the system.	2	5	4.37	0.70
B4		I can operate the interface of this system.	3	5	4.39	0.56
B1		I think it is easy to operate this system.	3	5	4.44	0.57
B10		I can clearly understand the feedback of this system.	3	5	4.19	0.64
B11		I feel the interaction of the system interesting.	3	5	4.30	0.63
B12	Efficiency (Group FB2)	I can use this system smoothly.	2	5	3.72	0.89
B6		I think this system has effectively integrated several functions.	2	5	4.03	0.77
B2		I cannot concentrate to use this system.	2	5	3.75	0.86
B9	Satisfaction (Group FB3)	I cannot utilize each item of the system.	2	5	4.13	0.69
B8		I think the feedback of this system is rich.	2	5	4.03	0.79
B5		This system fails to attract my attention.	2	5	4.01	0.73
B7		I like the way of presentations of this system.	3	5	4.43	0.56
**Average**					**4.19**	
C3	Affective or cognitive experience (Group FC1)	I am happy after using the interactive device of this system.	2	5	4.11	0.79
C5		The audio-visual feedback of the system can attract my attention.	2	5	4.24	0.64
C2		This system can help me control emotions better.	2	5	3.87	0.90
C7		I am more willing to know more about calligraphy after using the interactive system.	2	5	3.68	0.77
C8	Sensory experience (Group FC2)	I can feel the change of emotions from the feedback of the system after using the interactive device of this system.	1	5	4.26	0.77
C9		This system makes me aware of the importance of emotion expressions.	3	5	4.02	0.69
C1		The way of operations of the system can attract my attention.	3	5	4.33	0.58
C4	Social-identity experience or behavior (Group FC3)	This system changes my opinion on calligraphy.	2	5	4.06	0.80
C6		This system can help me understand better the meaning of calligraphy.	2	5	4.01	0.76
C10		I am more willing to experience interactive and scientific works after using the device of the interactive system.	3	5	4.45	0.57
**Average**					**4.10**	

**Table 15:** Cronbach’s α coefficients of the six question dimensions of the two indicators “system usability” and “writer’s experience” of the collected questionnaire data.

Indicator	Question Dimension (Scale)	Cronbach’s α Coefficient of the Question Dimension	Cronbach’s α Coefficient of the Indicator
System usability	Ease to learn	0.781	0.851
	Efficiency	0.671	
	Satisfaction	0.783	
Writer’s experience	Affective or cognitive experience	0.764	0.828
	Sensory experience	0.741	
	Social-identity experiences or behavior	0.643	

**Table 16:** Structure-model fit indices of the two indicators of “system usability” and “writer’s experience” yielded by the CFA process ^a^.

Scale	df	χ^2^	χ^2^/df	agfi	cfi	RMSEA	RMSEA (90% CI)	
							LO	HI
System usability	51	60.64	1.19	0.79	0.95	0.06	0.00	0.11
Writer’s experience	32	52.71	1.65	0.76	0.87	0.11	0.05	0.16

^a^ Meanings of symbols—df: degree of freedom; gfi: goodness-of-fit index; agfi: average gfi; cfi: comparative fit index; RMSEA: root mean square error of approximation; CI: confidence interval; LO: low; HI: high.

**Table 17:** Construct validity values of the question dimensions (scales) of the two indicators of “system usability” and “writer’s experience” yielded by the CFA.

Indicator	Question Dimension (Scale)	Group of Related Questions	Construct Validity Value
System usability	Ease to learn	FB1 = (B1, B3, B4, B10, B11)	0.773
	Efficiency	FB2 = (B2, B6, B12)	0.665
	Satisfaction	FB3 = (B5, B7, B8, B9)	0.784
Writer’s experience	Affective or cognitive experience	FC1 = (C2, C3, C5, C7)	0.775
	Sensory experience	FC2 = (C1, C8, C9)	0.745
	Social-identity experiences or behavior	FC3 = (C4, C6, C10)	0.653

**Table 18:** Average scores of the evaluation of the system usability from indicator viewpoints.

Indicator	N	Average Mean	Standard Deviation
Ease to learn	54	4.34	0.62
Efficiency	54	3.83	0.84
Satisfaction	54	4.15	0.69

**Table 19:** Percentage statistics of the evaluation of the system usability from indicator viewpoints.

Indicator	Strongly Agree	Agree	No Opinion	Disagree	Strongly Disagree
Ease to learn	41.84	50.38	7.42	0.36	0
Efficiency	20.37	52.47	17.90	9.27	0
Satisfaction	31.93	55.10	10.65	2.33	0

**Table 20:** Average scores of the evaluation of the writers’ experiences from indicator viewpoints.

Question Dimension	N	Average Mean	Standard Deviation
Affective or cognitive experience	54	4.22	0.78
Sensory experience	54	4.20	0.68
Social-identity experience or behavior	54	4.17	0.71

**Table 21:** Percentage statistics of the evaluation of the writers’ experiences from indicator viewpoints.

Question Dimension	Strongly Agree	Agree	No Opinion	Disagree	Strongly Disagree
Affective or cognitive experience	26.4	50.0	18.5	5.1	0.0
Sensory experience	33.3	55.0	10.5	0.6	0.6
Social-identity experience or behavior	36.4	45.7	16.7	1.2	0.0

**Table 22:** Interview results obtained from the writers participating in the formal exhibition.

Indicator	Question Asked in Interview	Record of Interviewee’s Opinions
Operation on man-machine interfacing	What do you think of the interaction based on the use of the brain wave?	Most writers thought the interaction with the brain wave machine novel and interesting. (21)* The writers had no special views on the use of the brain wave instrument in interaction. (3) A few writers believed that it was a bit strange to wear the brain wave instrument. (1)
	What do you think of write calligraphy on a touch screen?	The writers believed that the feedback was slow, and the touch screen can be improved. (5) The writers believed that the fluency of operations was better than the earlier system. (6) The writers thought that calligraphy on the touch screen was different from the traditional calligraphy experience. (8) The writers thought that writing on the touch screen was novel and environment-friendly as it did not need paper to write. (6)
	What do you think of the operation interface of this system?	Most writers thought that the overall operation interface was easy to understand. (19) Some writers said that they did not know what the purpose of the button of ‘send’ was. (4) A small number of writers misunderstood the sequence of “send” and “seal” in the interaction. (2)
Opinions on calligraphy writing	What do you think of the digital technique of expression of calligraphy writing, compared with the traditional calligraphy writing?	The writers asserted that this system had richer and more interesting feedback than traditional calligraphy and brought about relaxing feelings to them. (9) The writers believed that this work can provide an introductory experience for children or those who had not been exposed to calligraphy before. (8) The writers believed that although the digital way of calligraphy can save the process of preparing writing props, it reduced some experiences of traditional calligraphy. (5) The writers believed that the digital way can help them understand their emotional state when writing, rather than simply watch their written works. (3)
	Can this interactive system enhance your willingness to know more about calligraphy?	Most of the writers said that this system allowed them to recall the memory of learning calligraphy, and wanted to experience calligraphy further again. (19) Some writers thought that this system did not increase their willingness to re-engage in calligraphy. (5) A small number of the writers mentioned that they are familiar with calligraphy, so they had no special ideas. (1)
Opinions on affective computing	Can you feel your emotional change based on the feedback of the system?	Most of the writers could feel their emotions through the system feedback (sound playing and animation changes in the interaction pool). (20) Some writers mentioned that they could not notice the visual changes in the interaction pool when writing because of their concentration on writing. (5)
	Can you feel that emotions are related to calligraphy after you interacted with the system?	Most writers mentioned that in the process of interaction with the system, they could feel the interrelation between their emotions and writing actions. (21) Some writers pointed out that there was no correlation between their emotions and writing actions in the experiencing process. (4)

* Note: the number in parentheses means the number of writers giving the opinions.

**Figure A1:**
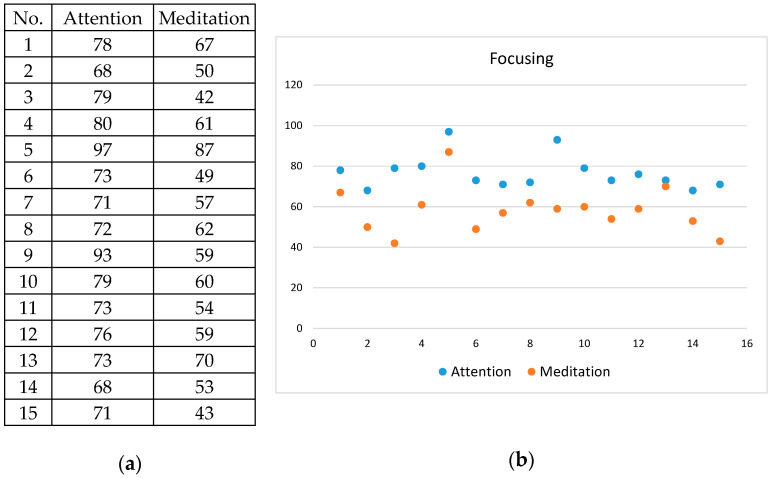
The data and the distribution graph of the average values of the 15 measured two-feature sample sequences of the emotion type of focusing, from which the corresponding decision rule *a* ≥ 70 and *a* ≥ *m* for emotion classification was inferred, where *m* and *a* represent the meditation and attention signals, respectively. (**a**) The average data. (**b**) The distribution graph.

**Figure A2:**
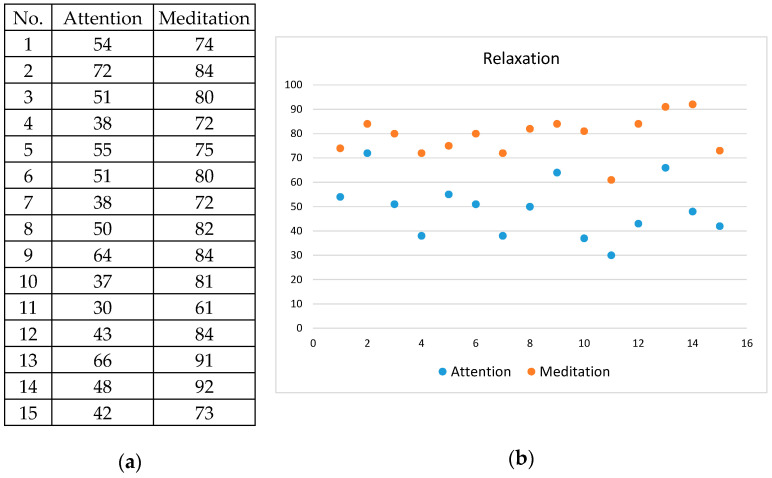
The data and the distribution graph of the average values of the 15 measured two-feature sample sequences of the emotion type of relaxation, from which the corresponding decision rule *m* ≥ 70 and *m* > *a* for emotion classification was inferred, where m and a represent the meditation and attention signals, respectively. (**a**) The average data. (**b**) The distribution graph.

**Figure A3:**
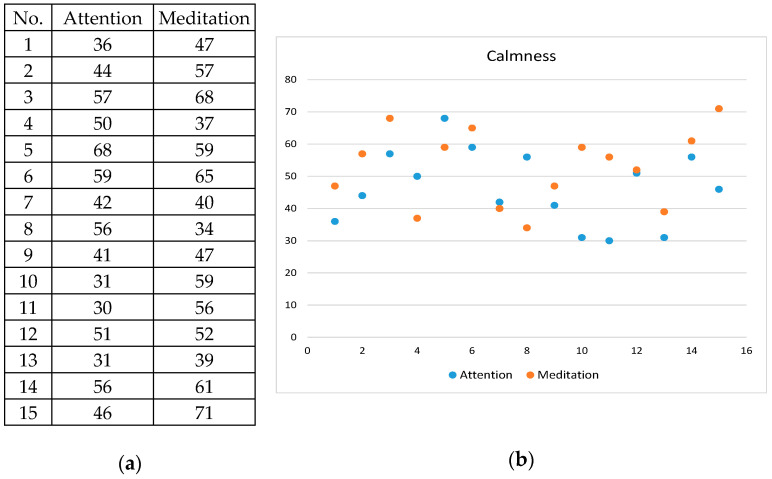
The data and the distribution graph of the average values of the 15 measured two-feature sample sequences of the emotion type of calmness, from which the corresponding decision rule 30 ≤ *a* < 70 and 30 ≤ *m* < 70 for emotion classification was inferred, where *m* and *a* represent the meditation and attention signals, respectively. (**a**) The average data. (**b**) The distribution graph.

**Figure A4:**
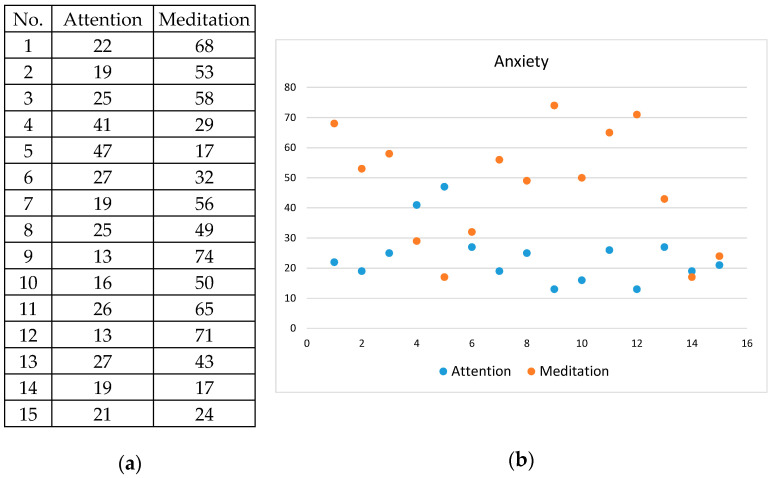
The data and the distribution graph of the average values of the 15 measured two-feature sample sequences of the emotion type of anxiety, from which the corresponding decision rule *a* < 30 or *m* < 30 for emotion classification was inferred, where *m* and *a* represent the meditation and attention signals, respectively. (**a**) The average data. (**b**) The distribution graph.

**Figure A5:**
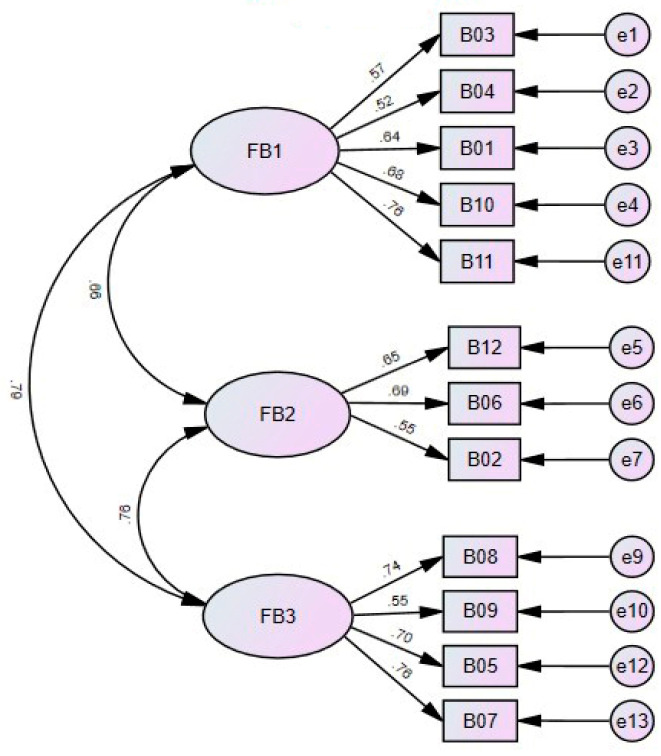
The 3-scale structure model graph yielded by the CFA of the indicator of “system usability” (FB1: ease to learn; FB2: efficiency; FB3: satisfaction).

**Figure A6:**
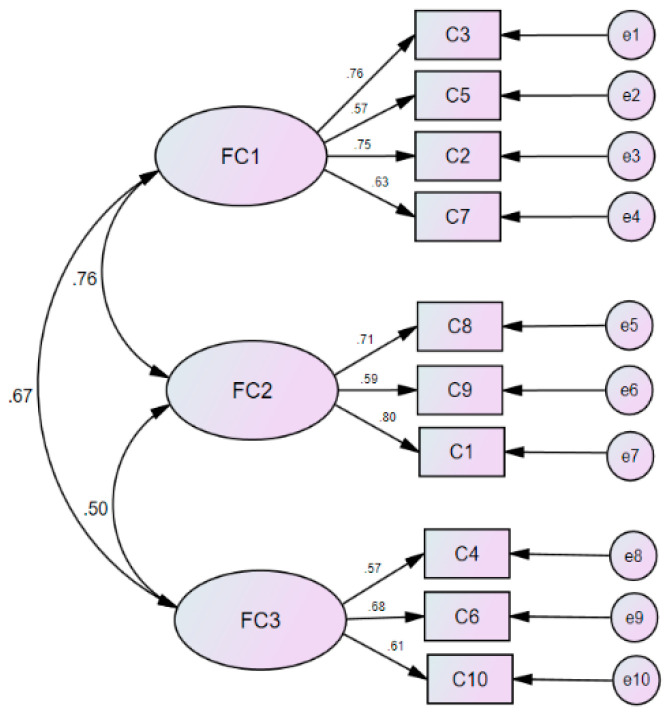
The 3-scale structure model graph yielded by the CFA of the indicator of “writer’s experience” (FC1: affective or cognitive experience; FC2: sensory experience; FC3: social-identity experience or behavior).

## Appendix A. Distribution Graphs of the Averages of the Sample Sequences of the Brain Wave Data of the Four Emotion Types

In this appendix, the distribution graphs of the 15 average values of the two-feature sample sequence data of attention and meditation of the four emotion types mentioned in Section 4.3.2 are shown. In each of Figure A1, Figure A2, Figure A3 and Figure A4, the data of attention and meditation are tabularized on the left and the corresponding graph is shown on the right.

## Appendix B. Finding the Latent Scales of Questions and Proving the Reliability and Validity of the Questionnaire Data

Listed in this appendix are the detailed results yielded by the uses of the SPSS and AMOS software packages to conduct various tests or analyses of the data shown in Table 11 and Table 12 in the main text, which were collected from the questionnaire surveys in the second experiment of this study.

### Appendix B.1. Testing Adequacy of Questionnaire Data by the KMO and Bartlett’s Tests

The results of the two tests yielded by the SPSS for the two indicators of system usability and writer’s experience, respectively, are shown in Table A1 and Table A2.

**Table A1:** Results of KMO and Bartlett’s Tests for the indicator of system usability.

Kaiser-Meyer-Olkin Measure of Sampling Adequacy		0.796
Bartlett’s Test of Sphericity	Approx. Chi-Square	228.865
	Degree of freedom	66
	Significance	0.000

**Table A2:** Results of KMO and Bartlett’s Tests for the indicator of writer’s experience.

Kaiser-Meyer-Olkin Measure of Sampling Adequacy		0.735
Bartlett’s Test of Sphericity	Approx. Chi-Square	185.107
	Degree of freedom	45
	Significance	0.000

### Appendix B.2. Finding Latent Question Dimensions (Scales) of the Questions from the Collected Data

(A) For the indicator of system usability

The SPSS was used to perform exploratory factor analysis (EFA) by principal component analysis and the method of varimax with Kaiser normalization to find suitable question dimensions (scales) for the originally designed questions using the collected questionnaire data. The original questions designed in this study with labels B1 through B12 are shown in the upper part of Table 6 in the main text. After the EFA was applied to the data in the table using principal component analysis and the varimax method with Kaiser normalization, a rotated component matrix was made, as shown in Table A3, from which it can be seen that the questions were re-arranged into three different groups FB1 = (B3, B4, B1, B10, B11), FB2 = (B12, B6, B2), and FB3 = (B9, B8, B5, B7) according to the clusters of large values of the respective scales found in the matrix. The three grouped are still named in this study by the titles of “ease to learn,” “efficiency,” and “satisfaction,” of the original three scales, respectively, as shown in Table A4.

(B) For the indicator of writer’s experience

Similar operations were conducted to the original questions labelled C1 through C12 of the second indicator of writer’s experience shown in the lower part of Table 6 in the main text. The result was a rotated component matrix, as shown in Table A5 below, from which it can be seen that the questions were also re-arranged into three different groups FC1 = (C7, C3, C5, C2), FC2 = (C8, C9, C1), and FC3 = (C4, C6, C10). The three grouped are named “affective or cognitive experience,” “sensory experience,” and “social-identity experience or behavior,” respectively, as shown in Table A6.

**Table A3:** Rotated component matrix.

Lebel	Question Dimension (Scale)		
	1	2	3
B3	**0.780**	0.025	0.104
B4	**0.727**	0.016	0.062
B1	**0.710**	0.147	0.196
B10	**0.595**	0.253	0.338
B11	**0.567**	0.501	0.251
B12	0.154	**0.771**	0.084
B6	0.256	**0.729**	0.099
B2	−0.115	**0.717**	0.151
B9	0.122	−0.038	**0.892**
B8	0.357	0.294	**0.672**
B5	0.119	0.474	**0.599**
B7	0.305	0.503	**0.523**
Extraction method: principal component analysis. Rotation method: varimax with Kaiser normalization. Rotation converged in 5 iterations.			

**Table A4:** Question dimensions (scales) and statistics of questionnaire data of the indicator of “system usability.”.

Label	Question Dimension	Question	Min	Max	Mean	S. D.
B3	Ease to learn (Group FB1)	I can quickly perform each step of using the system.	2	5	4.37	0.70
B4		I can operate the interface of this system.	3	5	4.39	0.56
B1		I think it is easy to operate this system.	3	5	4.44	0.57
B10		I can clearly understand the feedback of this system.	3	5	4.19	0.64
B11		I feel the interaction of the system interesting.	3	5	4.30	0.63
B12	Efficiency (Group FB2)	I can use this system smoothly.	2	5	3.72	0.89
B6		I think this system has effectively integrated several functions.	2	5	4.03	0.77
B2		I cannot concentrate to use this system.	2	5	3.75	0.86
B9	Satisfaction (Group FB3)	I cannot utilize each item of the system.	2	5	4.13	0.69
B8		I think the feedback of this system are rich.	2	5	4.03	0.79
B5		This system fails to attract my attention.	2	5	4.01	0.73
B7		I like the way of presentations of this system.	3	5	4.43	0.56
**Average**					**4.19**	

**Table A5:** Rotated component matrix.

Lebel	Question Dimension (Scale)		
	1	2	3
C7	**0.806**	0.082	0.127
C3	**0.775**	0.140	0.296
C5	**0.586**	0.400	−0.024
C2	**0.524**	0.432	0.348
C8	0.182	**0.868**	−0.106
C9	0.077	**0.754**	0.303
C1	0.518	**0.621**	0.081
C4	−0.002	0.075	**0.850**
C6	0.359	−0.045	**0.721**
C10	0.201	0.416	**0.521**
Extraction method: principal component analysis. Rotation method: varimax with Kaiser normalization. Rotation converged in 5 iterations.			

**Table A6:** Question dimensions (scales) and statistics of questionnaire data of the indicator of “writer’s experience.”.

Label	Question Dimension	Question	Min	Max	Mean	S. D.
C3	Affective or cognitive experience (Group FC1)	I am happy after using the interactive device of this system.	2	5	4.11	0.79
C5		The audio-visual feedback of the system can attract my attention.	2	5	4.24	0.64
C2		This system can help me control emotions better.	2	5	3.87	0.90
C7		I am more willing to know more about calligraphy after using the interactive system.	2	5	3.68	0.77
C8	Sensory experience (Group FC2)	I can feel the change of emotions from the feedback of the system after using the interactive device of this system.	1	5	4.26	0.77
C9		This system makes me aware of the importance of emotion expressions.	3	5	4.02	0.69
C1		The way of operations of the system can attract my attention.	3	5	4.33	0.58
C4	Social-identity experience or behavior (Group FC3)	This system changes my opinion on calligraphy.	2	5	4.06	0.80
C6		This system can help me understand better the meaning of calligraphy.	2	5	4.01	0.76
C10		I am more willing to experience interactive and scientific works after using the device of the interactive system.	3	5	4.45	0.57
**Average**					**4.10**	

### Appendix B.3. Verifying the Reliability of the Collected Data by the Cronbach’s α Coefficients Yielded by the EFA

The Cronbach’s α coefficient values yielded by the EFA of the three question dimensions (scales) of “ease to learn,” “efficiency,” and “satisfaction” of the indicator of system usability are shown in Table A7; and those of the three question dimensions (scales) of “affective or cognitive experience,” “sensory experience,” and “social-identity experience or behavior” of the indicator writer’s experience are shown in Table A8. It can be seen from the two tables that the coefficient values of the six scales and those of the two indicators are all in the range of 0.35 to 0.70 or even larger, meaning that the collected questionnaire data are reliable, because according to Gilford [50], the goodness of the reliability of collected data can be judged from the following rules:

$$\begin{array}{lll} \alpha \geq 0.70 & \to & \mathrm{highly}\;\mathrm{reliable};\\ 0.35\leq \alpha <0.70 & \to & \mathrm{reliable};\\ \alpha \leq 0.35 & \to & \mathrm{unreliable}, \end{array}$$

**Table A7:** Cronbach’s α coefficients of the three question dimensions of the indicator “system usability” of the collected questionnaire data.

Indicator	Question Dimension (Scale)	Cronbach’s α Coefficient of the Question Dimension	Cronbach’s α Coefficient of the Indicator
System usability	Ease to learn	0.781	0.851
	Efficiency	0.671	
	Satisfaction	0.783	

**Table A8:** Cronbach’s α coefficients of the three question dimensions of the indicator “writer’s experience” of the collected questionnaire data.

Indicator	Question Dimension (Scale)	Cronbach’s α Coefficient of the Question Dimension	Cronbach’s α Coefficient of the Indicator
Writer’s experience	Affective or cognitive experience	0.764	0.828
	Sensory experience	0.741	
	Social-identity experience or behavior	0.643	

### Appendix B.4. Verifying the Suitability of the Model Structure of the Data Set Up by the Question Dimensions

(A) Analysis for the indicator of system usability

The result of performing the confirmatory factor analysis (CFA) process using the AMOS package on the questionnaire data of the indicator of system usability shown in Table A4 is a 3-scale structure-model graph, as shown in Figure A5; and a list of structure-model fit indices yielded by the CFA, including the degree of freedom (df), the chi-square (χ^2^) statistics, the ratio of χ^2^/df, the adjusted goodness-of-fit index (gfi), the comparative fit index (cfi), and the root mean square error of approximation (RMSEA), are shown in Table A9.

**Table A9:** Structure-model fit indices of the indicator of “system usability” yielded by the CFA process ^a.^

Index	df	χ^2^	χ^2^/df	agfi	cfi	RMSEA	RMSEA (90% CI)	
							LO	HI
Value	51	60.64	1.19	0.79	0.95	0.06	0.00	0.11

^a^ Meanings of symbols—df: degree of freedom; gfi: goodness-of-fit index; agfi: average gfi; cfi: comparative fit index; RMSEA: root mean square error of approximation; CI: confidence interval; LO: low; HI: high.

From Table A9, we have the following observations: (1) the small value, 1.19, of χ^2^/df (the ratio the χ^2^ value to the degree of freedom df) means that the model is of a good fit because a value of χ^2^/df smaller than 3 and larger than 1 means the model is of a good fit according to Hair et al. [53]; (2) the agfi value of 0.79 means that the 3-scale model structure of the adopted questionnaire is reasonably good, because when the sample number is not large as the case of this study, the threshold for judging the goodness of fit of the model may be taken to be approximately 0.8 according to MacCallum and Hong [54]; (3) the cfi value of 0.95 shows that the model is good in structure because a cfi value around 0.90 or larger means that the model has a reasonable or even better goodness of fit according to Bentler [55]; (4) the RMSEA value of 0.06 means that the model has a reasonable goodness of fit, because according to Hu and Bentler [56], an RMSEA value no larger than 0.06 means that the model has a good goodness of fit to the collected data. All these four facts point out that the structure of the 3-scale model set up by the question dimensions of the indicator of system usability described previously has a reasonable goodness of fit to the collected questionnaire data.

(B) Analysis for the indicator of writer’s experience

The result of performing the CFA process on the questionnaire data of the indicator of writer’s experience shown in Table A6 is also a 3-scale structure-model graph, as shown in Figure A6; and a list of structure-model fit indices yielded by the CFA like those shown in Table A9 is shown Table A10. From the table, it can be seen that all the three values of χ^2^/df, agfi, and cfi show that the structure model set up by the question dimensions (scales) has a good fit to the collected data according to similar analyses to those conducted for the indicator of system usability. An exception is the RMSEA value of 0.11, which is larger than the threshold 0.10 mentioned previously. However, as mentioned in Fan et al. [57], when the sample size is small as the case in this study, the RMSEA value will increase. Therefore, the RMSEA value of 0.11 here, which is a little bit larger than the threshold 0.11, may still be acceptable.

In short, like the case of the indicator of system usability, all the four index values mentioned above point out that the 3-scale model structure set up by the question dimensions of the indicator of writer’s experience described previously has a reasonable goodness of fit to the collected questionnaire data.

**Table A10:** Structure-model fit indices of the indicator of “writer’s experience” yielded by the CFA process ^a.^

Index	df	χ^2^	χ^2^/df	agfi	cfi	RMSEA	RMSEA (90% CI)	
							LO	HI
Value	32	52.71	1.65	0.76	0.87	0.11	0.05	0.16

^a^ Meanings of symbols—df: degree of freedom; gfi: goodness-of-fit index; agfi: average gfi; cfi: comparative fit index; RMSEA: root mean square error of approximation; CI: confidence interval; LO: low; HI: high.

### Appendix B.5. Proving the Validity of the Questionnaire Data Using the Data Yielded by the EFA and CFA

With the model structures of the two indicators both proven to have reasonable goodness of fit to the collected questionnaire data, it is appropriate to analyze further the validity of the data.

(A) Analysis for the indicator of system usability

It can be seen from the 3-scale structure model shown in Figure A5 that all the factor loading values (also called standardized regression weights) with respective to the scales (appearing on the paths from the scales FB1–FB3 to the questions B01–B12) are all larger than the threshold of 0.5, meaning that the construct validity of the model was secured; and this fact can be proved equivalently by the construct validity values of all the three scales of the indicator of system usability yielded in the EFA process using the SPSS, which are listed in Table A11 and can be seen to be all larger than the threshold value of 0.6 [58,59]. That is, the validity of the collected questionnaire data of the indicator of system usability is proved.

**Table A11:** Construct validity values of the three scales of the indicator of “system usability.”.

Scale	Group of Related Questions	Construct Validity Value
Ease to learn	FB1 = (B1, B3, B4, B10, B11)	0.773
Efficiency	FB2 = (B2, B6, B12)	0.665
Satisfaction	FB3 = (B5, B7, B8, B9)	0.784

(B) Analysis for the indicator of writer’s experience

The two facts mentioned above about the data validity of the indicator of system usability can also be found in the data of the indicator of writer’s experience. That is, all the factor loading values with respect to the scales on the 3-scale structure model shown in Figure A6 are all larger than the threshold of 0.5, and the construct validity values of the three scales yielded in the EFA as listed in Table A12 are all larger than the threshold of 0.6 [58,59].

**Table A12:** Construct validity values of the three scales of the indicator of “writer’s experience”.

Scale	Group of Related Questions	Construct Validity Value
Affective or cognitive experience	FC1 = (C2, C3, C5, C7)	0.775
Sensory experience	FC2 = (C1, C8, C9)	0.745
Social-identity experiences or behavior	FC3 = (C4, C6, C10)	0.653
